# Recyclable Li‐Metal Battery Electrolytes via In Situ Cyclic Carbonate Polymerization

**DOI:** 10.1002/advs.202504206

**Published:** 2025-06-09

**Authors:** Hui Gao, Victor Riesgo‐Gonzalez, James R. Runge, Kanyapat Yiamsawat, Dominic Spencer‐Jolly, Thomas M. McGuire, Gregory J. Rees, Xiangwen Gao, Bingkun Hu, Shengming Zhang, Longlong Wang, Peter G. Bruce, Georgina L. Gregory, Charlotte K. Williams

**Affiliations:** ^1^ Chemistry Research Laboratory University of Oxford Oxford OX1 3TA UK; ^2^ Department of Materials University of Oxford Oxford OX1 3PH UK; ^3^ The Faraday Institution Quad One Harwell Science and Innovation Campus Didcot OX11 0RA UK

**Keywords:** batteries, in situ polymerizations, lithium metal, polymer electrolytes, recycling

## Abstract

Enabling recycling and improving performance are key challenges for next‐generation electrolytes for rechargeable batteries. Here, an equilibrium polymerization: trimethylene carbonate (TMC) ring‐opening polymerization, in the presence of lithium difluoro(oxalato)borate salt, is utilized to form an electrolyte in situ during coin cell fabrication for lithium batteries. This process creates a semi‐solid poly(trimethylene carbonate) electrolyte with high ambient ionic conductivity (0.52 mS cm^−1^), thermal stability (*T*_d, 5%_ = 160 °C), and oxidative stability up to 4.7 V. Using this electrolyte with commercial lithium iron phosphate cathodes, results in 97% capacity retention after 350 cycles at 2C, achieving theoretical capacities of 170 mAh g^−1^ at 0.1C. The cells retain excellent performance at high current densities (86 mAh g^−1^ at 4C). Post‐use, the polymer can be separated from the salt and selectively recycled to pure starting monomer (TMC) through a solid‐state chemical recycling process. The recycled monomer, when repolymerized to reform the polycarbonate electrolyte, yields cells with performance identical to the original. The exploitation of polymerization‐depolymerization equilibria offers a useful strategy for enhancing battery performance, ensuring effective material recycling, and advancing a circular economy.

## Graphical abstract

A recyclable polycarbonate electrolyte is synthesized in situ in a lithium‐metal battery. Excellent cell performance is obtained owing to its high conductivity and (electro)chemical stability. The electrolyte is recovered after extended battery cycling, chemically recycled back to monomer, and repolymerized, obtaining comparable cell performance. This concept will help develop future recyclable polymer electrolytes and deliver a circular economy for batteries.

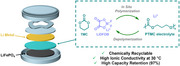

## 1 Introduction

The lithium‐ion battery has become essential to modern life, powering portable electronics, facilitating the electric transportation sector, and enabling large‐scale grid energy storage.^[^ ^1^ ^]^ With battery production set to increase exponentially in the coming years,^[^ ^2^ ^]^ it is essential to assess the environmental impact of materials, production, and recycling early on to align with circular economy principles.^[^ ^3^ ^]^ As such, there is growing interest in simplifying the manufacture of batteries to ensure that the next generation of lithium batteries are designed with recycling in mind.^[^ ^4^ ^]^ At the same time, increasing energy density and safety are vital to meet the growing energy storage demands, particularly in transportation.^[^ ^5^ ^]^ One promising route toward this end is replacing flammable liquid electrolytes with solid‐state counterparts that can mitigate safety issues due to reduced flammability and may allow the use of metallic anodes such as lithium metal in the future.^[^ ^6^ ^]^ Some inorganic ceramic solid electrolytes, *e.g*. Li_6_PS_5_Cl, have demonstrated high ionic conductivities (> 1 mS cm^−1^),^[^ ^7^ ^]^ but their air sensitivity complicates processing and prevents straightforward material re‐manufacturing and recycling.^[^ ^8^ ^]^ In contrast, polymer electrolytes possess mechanical properties facilitating interfacial contact and charge transport with electrodes, particularly at low temperatures and low stack pressures.^[^ ^9^ ^]^ On the other hand, examples of polymer electrolytes that are truly stable against Li metal and suppress Li dendrite growth are rare.^[^ ^10^ ^]^ Polymer electrolytes may also help to improve cell sustainability by simplifying manufacturing processes, reducing embedded energy, and enabling effective, low‐energy recovery, reuse, and recycling after end‐of‐life.^[^ ^9^ ^]^ Effective removal of the electrolyte may also help to separate the valuable inorganics and metals used in rechargeable batteries.

Battery recycling is essential to reduce the ecological footprint and overcome future shortages of critical metals and minerals used as raw materials.^[^ ^11^ ^]^ Process chains for battery recycling and reconditioning are still under development, but many governments and international authorities have set ambitious targets for battery recycling. Currently, industrialized battery recycling is pyrometallurgical, i.e. pyrolysis at temperatures >1500 °C.^[^ ^12^ ^]^ Academic research into battery recycling to try to recover metals under lower temperature conditions typically requires very toxic solvents like *N*‐methyl‐2‐pyrrolidone (NMP) or dimethylformamide (DMF) to remove the polymer.^[^ ^13^ ^]^ This work focuses on developing chemically recyclable polymer electrolytes. Our approach is to exploit polymerization/depolymerization thermodynamics to target polymer electrolytes, which are straightforward to prepare and can be recycled to monomer after use.^[^ ^14^ ^]^ Whilst there have been previous reports targeting degradable polymer electrolytes,^[^ ^15^ ^]^ there are no prior studies exploiting the polymerization equilibrium for recycling. For example, a fluorinated polyester electrolyte demonstrated degradability after use in the battery, by base‐catalysed hydrolysis, to form mixtures of di‐acid and diol small molecules. These monomers were repolymerized, with extrusion of an equivalent of water per repeat unit of the polymer chain, and with freshly added fluorinated end‐capping molecules to form a new polymer electrolyte.^[^ ^15c^ ^]^ Another concept is to design polymer electrolytes featuring dynamic bonds which can be triggered to facilitate self‐healing and re‐processing; some of these dynamic polymers were used in batteries, but performances remained rather modest in terms of capacity and capacity retention over repeated cycles.^[^ ^16^ ^]^ One limitation of current polymer recycling strategies is that they either produce water during repolymerization or result in the exposure of hydrophilic polymers; indeed, processes introducing water into the recycled cell would be a major risk to the moisture sensitive inorganics including, the lithium anode, lithium salts, many cathodes (e.g., LiNi_x_Mn_y_Co_z_O_2_) and to any ceramic electrolytes (e.g. Li_6_PS_5_Cl).

An attractive but unexplored strategy in polymer electrolyte recycling is to exploit equilibrium ring‐opening polymerizations (ROP) to control polymer formation (battery manufacture) and depolymerization to monomer (battery recycling). These are addition polymerizations so obviate water or any other small‐molecule by‐product formation and, with the right catalyst, they can be very well controlled. Heterocycle ROP is already widely used to make polymer electrolytes, from the well‐known ethene oxide ROP to make the ubiquitous poly(ethylene oxide) (PEO). It has even been used to make polymer electrolytes in situ during battery fabrication;^[^ ^17^ ^]^ such in situ processes should improve electrode wetting by the liquid precursors, simplify cell manufacturing, and improve polymer‐electrode interfacial contact.^[^ ^18^ ^]^ Another advantage of in situ heterocycle ring‐opening polymerization is the ability to control the monomer/polymer conversion, a feature that is difficult with conventional condensation polymerizations. This allows for control of residual monomer content and degree of plasticization to achieve desired mechanical properties, high ionic conductivity, and optimized battery performance, while addressing the safety concerns associated with liquid electrolytes.^[^ ^17a^ ^]^ There is a particularly strong opportunity for semi‐solid electrolytes, which are defined as those where polymer (solid‐phase) is > 50 wt.%, as opposed to gel polymer electrolytes, in which the liquid fraction exceeds 50 wt.%.^[^ ^17a^ ^]^ There is already good precedence for in situ heterocycle ring‐opening polymerization to make semi‐solid polyethers,^[^ ^18^, ^19^ ^]^ polycarbonates,^[^ ^20^ ^]^ polyether‐*block‐*polycarbonates,^[^ ^21^ ^]^ and polyether‐*block*‐polyesters.^[^ ^22^ ^]^ One of the leading materials is poly(1,3‐dioxolane) (PDOL), produced by in situ cyclic ether ROP within solid‐state batteries.^[^ ^18^, ^19^ ^]^ The leading in situ PDOL solid and semi‐solid electrolytes combined low‐temperature ionic conductivity values > 1 mS cm^−1^, with promising cell cycling capabilities at practical C‐rates.^[^ ^19a^ ^]^ So far, there have not been any studies of the depolymerization, or closed‐loop chemical recycling, of these PDOL in situ electrolytes, perhaps due to their low thermal stability.^[^ ^23^ ^]^

We target a different approach by selecting a suitable heterocycle for ring‐opening that can be used to produce a semi‐solid electrolyte but also to allow for its efficient, low‐energy recycling to cyclic monomer.^[^ ^24^ ^]^ The polymerization thermodynamics control whether the polymer or monomer is favored under a particular set of conditions; the Gibbs free energy of polymerization depends upon the precise monomer/polymer chemical structure (ring‐size), nature, and sights for any substituents and chain end‐groups. For a particular monomer/polymer chemistry, the thermodynamics can be controlled by manipulating external conditions (monomer concentration/solvation, and temperature).^[^ ^25^ ^]^ In this work, we selected a 6‐membered ring‐derived aliphatic polycarbonate where ROP is enthalpically controlled, these polymers should favour recycling, by depolymerization, using higher temperatures or dilutions, the former being very much more attractive from a process/operability point of view.^[^ ^26^ ^]^ There are several challenges in applying these concepts: 1) The polycarbonate electrolytes should be prepared using cyclic carbonate ring‐opening polymerization in the presence of the lithium salt and in situ during battery fabrication; 2) The resulting polycarbonate electrolyte should show high conductivity and stability, including to any temperature increases which may occur during battery use; 3) the polycarbonate should be fully depolymerized to cyclic carbonate after use, and the cyclic monomer repolymerized to make a new batch of polymer electrolyte. We selected a commercial 6‐membered cyclic carbonate, trimethylene carbonate (TMC), and lithium difluoro(oxalato)borate (LiDFOB) as the salt. Trimethylene carbonate (TMC) is a six‐membered ring analog of the widely used five‐membered ring propylene carbonate, a common liquid electrolyte in lithium‐ion batteries. The key difference to propylene carbonate is that TMC has sufficient ring‐strain (enthalpy gain) to be polymerizable, however, its depolymerization equilibria can be readily controlled to re‐form the cyclic carbonate, under appropriate reaction conditions.^[^ ^26b^ ^]^ LiDFOB is a commonly used lithium borate salt, chosen due to its relatively high thermal stability, tendency to form a uniform solid electrolyte interface (SEI) at lithium metal anodes, and its precedent for passivation of aluminium current collectors.^[^ ^27^ ^]^

## 2 Results

### 2.1 In Situ Polycarbonate Electrolyte by Reversible Polymerization

**Figure 1:**
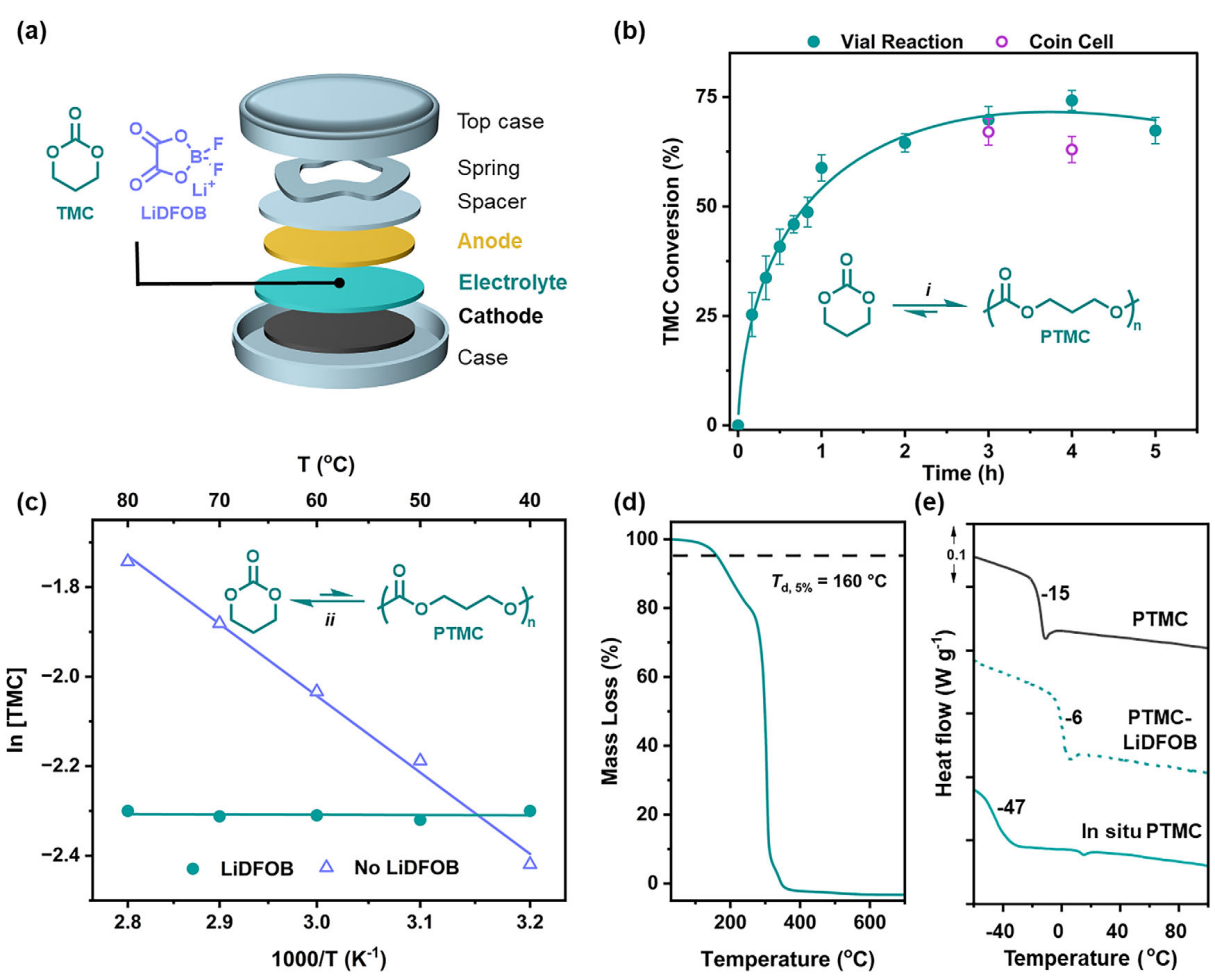
In situ PTMC electrolyte formation and monomer/polymer equilibrium. a) Schematic of coin cell showing TMC monomer and lithium salt structures. b) TMC ROP kinetics; *i* = 30 °C, P_2_‐*t*Bu catalyst (6 mol%), LiDFOB (10 mol%). TMC conversion was determined by ^1^H NMR spectroscopy (CDCl_3_) of aliquots quenched at indicated time points and determined for disassembled coin cells and model reactions conducted in a vial (see Supporting Information for experimental details). c) Change in TMC/PTMC conversion with heating (40–80 °C) and dilution (0.5 m in ACN) for ROP conducted in the presence and absence of LiDFOB (see Figure S9, Supporting Information for Δ*H*, Δ*S* values, and reaction conditions). d) TGA trace of in situ polymerized PTMC electrolyte. e) DSC thermograms of neat PTMC with and without LiDFOB compared to formed in situ PTMC electrolyte.

First, it was important to qualitatively understand how the in situ (battery manufacturing) conditions influence the polymerization, as such reactions were conducted in vials at 30 °C without stirring. The monomer, trimethylene carbonate (TMC), is a crystalline solid, but it melts in contact with LiDFOB (10 mol% vs. monomer) to form a homogeneous liquid solution. Polymerizations were conducted using 6 mol % vs. monomer P_2_‐*t*Bu (6:10:100 molar ratios of the P_2_‐*t*Bu:LiDFOB:TMC) with regular removal of aliquots, which were quenched, allowing the monomer conversion to be followed over time (**Figure** **1**b; Scheme S1, Supporting Information shows a proposed mechanism/catalytic cycle). Over 2 h, TMC conversion increased exponentially until it reached ≈63%, thereafter, it did not increase further. These data helped inform us upon the in situ PTMC semi‐solid electrolyte formation; accordingly, we designed the in‐cell process to involve first the addition of the liquid monomer (TMC) and lithium salt (LiDFOB) solution to the glass fibre separator used in a commercial coin cell. Subsequently, the liquid polymerization catalyst was added to the separator, noting that polymerization will begin as soon as the catalyst contacts the monomer (Figure 1a). The glass fibre separator was immediately placed between the lithium metal anode and LFP cathode, inside a resting coin cell at 30 °C, and the reaction was allowed to proceed for 4 h to reach equilibrium. The coin cell was disassembled after 4 h, and the resulting semi‐solid electrolyte was extracted and analyzed using ^1^H NMR spectroscopy. The polymerization achieved very similar conversion (63% after 4h, Figure 1b) to the reaction conducted in the vial, and the resulting polymer, analyzed by size exclusion chromatography (SEC), has a molar mass (*M*_n_) of 10.5 kg mol^−1^ with a dispersity (*Ð*) of 3.21 (Figure S5, Supporting Information). In conducting the proof‐of‐principle in situ polymer electrolyte investigation, it is pertinent to note that the times needed to make the PTMC electrolytes are considerably shorter than those equivalent in situ produced polymers, where up to 5 days can be necessary to produce semi‐solids due to slower reaction rates.^[^ ^21a^ ^]^

Considering the catalytic cycle, it is expected that the cyclic carbonate ROP produces polycarbonates with an alcohol (or alkoxide) chain end group (Scheme S1, Supporting Information). This functional group initiates the polymer/monomer equilibrium and enables chemical recycling.^[^ ^12^ ^]^ Nonetheless, such end groups might present compatibility issues with the cathode.^[^ ^21a^ ^]^ The monomer (TMC) / polymer (PTMC) electrolyte equilibrium thermodynamics were assessed to inform upon the influence of temperature on the equilibrium. If the polymer (PTMC) alcohol chain end group is active, heating and/or dilution should favor the formation of the monomer (TMC). For the PTMC polycarbonate electrolyte (i.e., with LiDFOB), neither extensive dilution in acetonitrile (0.1 m polymer solution) nor heating from 40–80 °C produced any significant change in conversion, as confirmed by ^1^H NMR spectroscopy (Figure 1c). In contrast, under the same conditions, the pure PTMC polymer underwent the expected depolymerization, and Van ‘t Hoff analysis allowed for the determination of the polymerization enthalpy and entropy values (Figure 1c). Furthermore, pure TMC ROP, under the same temperature and catalyst loadings, resulted in >99% conversion. These observations imply that the lithium salt, LiDFOB, is responsible for quenching the catalyst and/or alcohol polymer chain end group, limiting the depolymerization in the electrolyte. Given that the in situ TMC ROP reproducibly reached ≈60% conversion, it is proposed that such quenching/side reactions occurred competitively but more slowly than propagation. To investigate further, multi‐nuclear NMR spectroscopy experiments (^11^B, ^19^F, and ^31^P) were performed using mixtures of the lithium salt, phosphazene base catalyst, and an alcohol (as a model for the polymer chain end). Overall, these experiments imply that the lithium salt does not react significantly with the catalyst alone, but once an alcohol is present, there may be salt exchange processes which result in polymer chain end quenching, which might stop further polymerization conversion (Figures S2–S4, Supporting Information). In fact, a recent study has shown that boron containing species can inhibit the rate of cyclic ester ROP.^[^ ^28^ ^]^

The thermal stability of the in situ PTMC electrolyte was evaluated by thermogravimetric analysis (TGA) showing an onset of thermal degradation at 160 °C (*T*_d, 5%_, Figure 1d), confirming its improved stability compared to currently used liquid cyclic carbonate electrolytes, e.g. PC or EC, which begin to decompose from 80–100 °C.^[^ ^29^ ^]^ The semi‐solid electrolyte contains both the polymer (≈60%) and TMC monomer (40%). To further understand its thermal properties, differential scanning calorimetry (DSC) was conducted (Figure 1e). The in situ formed polymer electrolyte is amorphous and shows a glass transition temperature (*T*_g_) at –47 °C. In contrast, the pure PTMC polymer has a higher glass transition temperature at –15 °C whilst a PTMC electrolyte formed by the addition of LiDFOB after polymerization exhibited a *T*_g_ of –6 °C. The presence of residual TMC monomer in the in situ PTMC electrolyte has a plasticising effect on the polymer, greatly lowering the *T*_g_ of the semi‐solid polymer electrolyte. (Figure 1e). Both DSC and X‐ray diffraction (XRD) were used to confirm that the in situ PTMC electrolyte is amorphous (Figure S8, Supporting Information). For polymer electrolytes, the combination of low *T*_g_ and an amorphous structure is known to result in enhanced Li‐ion conductivity.^[^ ^9a^ ^]^

### 2.2 Mechanical Stability to Prevent Lithium Dendrites

**Figure 2:**
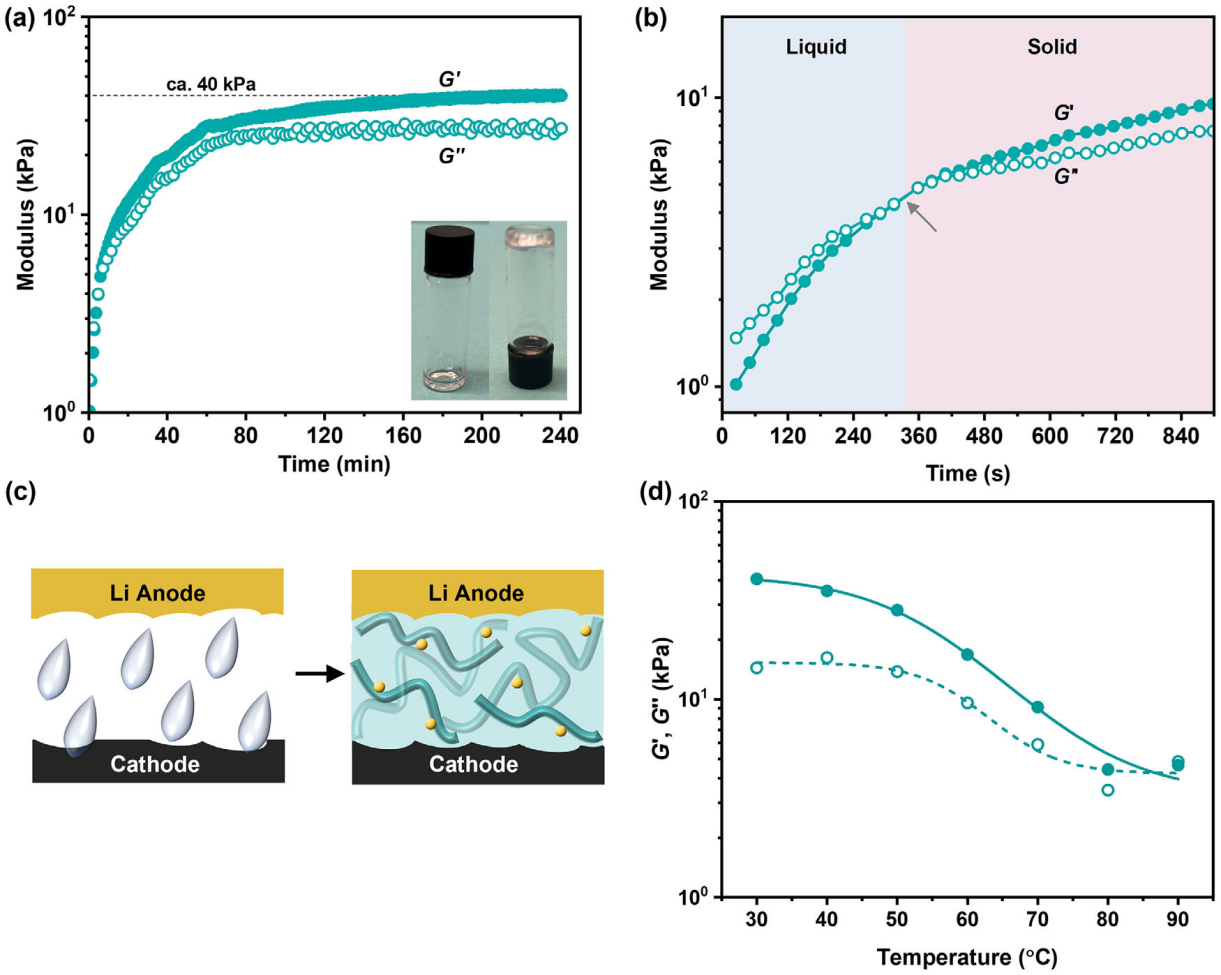
Rheological measurements of semi‐solid PTMC electrolyte. a) Change in shear loss (*G*’’) and storage (*G*’) moduli with time as ROP of TMC proceeds. Inset: photo of liquid precursor and resulting PTMC electrolyte after 4 h. b) Magnified view of liquid to solid‐like behavior showing the *G*’‐*G*’’ transition point. c) Schematic of electrolyte‐electrode interfaces indicating wetting of electrode surfaces by liquid precursor and cementing of interfaces in semi‐solid electrolyte. d) Viscoelastic properties as a function of temperature (1 rad s^−1^).

During the in‐cell electrolyte formation reaction, the materials’ properties change from liquid to semi‐solid (**Figure** **2**). To quantify these changes, the polymerization was measured by oscillatory shear rheology measurements. In the experiments, the starting monomer, lithium salt, and catalyst mixture were placed between the rheometer plates, and the reaction progress was monitored under shear (strain γ = 1%, angular frequency 1 rad s^−1^, and 30 °C). During the first 5 min of the reaction, the material's shear loss modulus (*G*’’) is greater than its storage modulus (*G*’), consistent with the starting material being a liquid. As the polymerization proceeds, the elastic modulus (*G*’) increases and crosses the viscous modulus (*G*’’), signalling the rheological transition to a semi‐solid (Figure 2a,b). This crossover occurs consistent with the formation of a free‐standing solid PTMC electrolyte. The crossover point, which is observed after only 5 min, suggests that low concentrations of PTMC are sufficient to cause gelation of the in situ electrolyte (*≈*10% conversion Figure 1b). The influence of temperature was also investigated using rheology, showing that the electrolyte remained as a semi‐solid (*G*’ > *G*’’) over the range 30‐70 °C but that it softens as highlighted by a reduction in the storage (elastic, *G*’) modulus from 40 to 5 kPa (Figure 2d), demonstrating reasonable mechanical stability of the semi‐solid PTMC electrolyte over this temperature range.

### 2.3 High Ionic Conductivity and Electrochemical Stability

**Figure 3:**
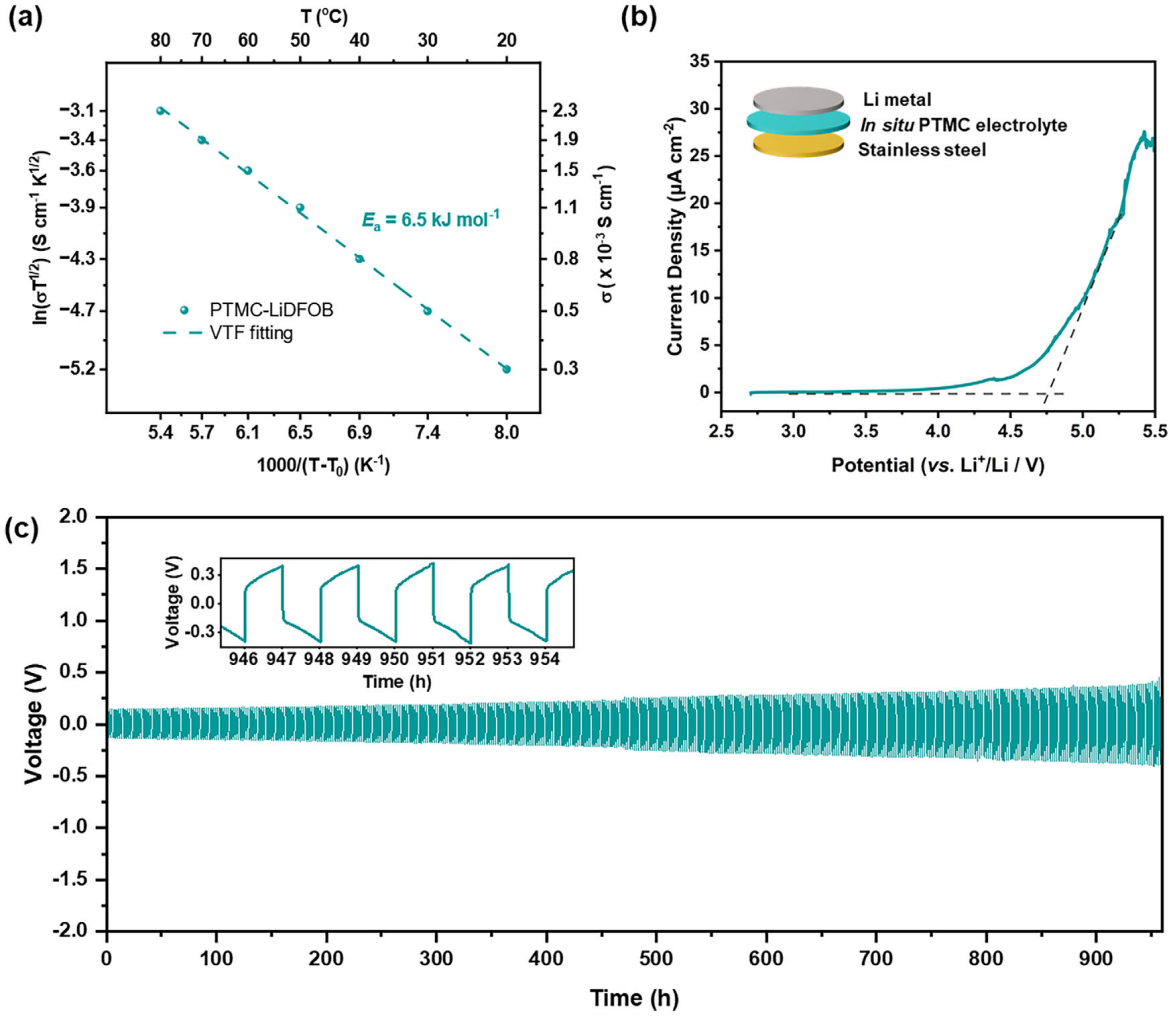
Ionic conductivity and electrochemical stability of PTMC electrolyte. a) Ionic conductivity as a function of temperature for in situ PTMC electrolyte; *T*_0_ = *T*_g_–50 K. b) Linear sweep voltammetry measured at 0.1 mV s^−1^ using lithium metal as counter/reference and stainless steel as working electrode. c) Long‐term cycling of a Li symmetric cell at a current density of 0.5 mA cm^−2^ with an areal capacity of 0.5 mAh cm^−2^.

The electrochemical properties of the in situ PTMC electrolyte were assessed since these are essential for any battery deployment. High ionic conductivity is needed to maintain practical charge‐discharge rates, and the electrolyte must remain stable alongside both the cathode and, ideally, lithium metal anode. Electrochemical impedance spectroscopy (EIS) was performed using two stainless steel blocking electrodes to measure the ionic conductivity of the semi‐solid PTMC electrolyte. At 30 °C, the in situ polycarbonate electrolyte demonstrated an impressive high ionic conductivity of 0.52 mS cm^−1^. This value is ≈10^2^–10^5^ times higher than the conductivity of related PTMC/Li‐salt electrolytes (10^−3^–10^−5^ mS cm^−1^ at 25 °C),^[^ ^30^ ^]^ underscoring the importance of the partial conversion and residual TMC monomer in facilitating lithium‐ion migration, through polymer plasticisation by the residual monomer (see Figure 1e). The temperature dependence of the ionic conductivity was determined and fit using the Vogel–Tammann–Fulcher (VTF) equation; it shows a low activation energy of 6.5 kJ mol⁻¹, consistent with high lithium‐ion mobility (**Figure** **3**a, Equation S1, Supporting Information). The ability to fit the conductivity data to the VTF equation supports that the polycarbonate (PTMC) segmental motion is the dominant ion‐transport mechanism rather than migration through the residual TMC monomer.

The anodic stability and compatibility of the in situ PTMC electrolyte were evaluated through linear sweep voltammetry (LSV) measurements and prolonged galvanostatic cycling in a symmetric lithium cell (Figure 3b,c). The results show that the electrolyte is oxidatively stable against stainless steel up to 4.7 V vs. Li/Li^+^, even at a slow scan rate of 0.1 mV s^−1^ (Figure 3b). Prolonged galvanostatic cycling in symmetric Li cells revealed stable performance over 960 h at a current density of 0.5 mA cm^−2^ and an areal capacity of 0.5 mAh cm^−2^, with only a 250 mV increase in voltage polarization (Figure 3c). Whilst promising, the observed polarization at low current densities and using thick Li metal electrodes (commonly used in the literature for in situ polymer electrolytes) suggests some degree of degradation or Li dendrite growth is taking place.^[^ ^19a^ ^]^ Nevertheless, a critical current density test up to 2.0 mA cm^−2^ for 35 h was successfully performed without short‐circuiting, despite significant polarization (Figure S12, Supporting Information), suggesting that in situ PTMC could be used at practical rates in Li metal batteries.

### 2.4 Long‐Term Cycling Performance in Lithium‐Metal Cells

**Figure 4:**
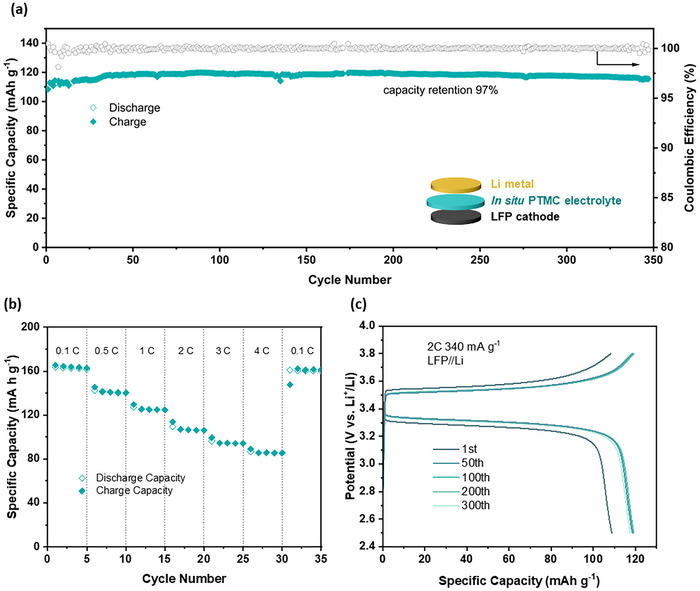
Cycling performance in Li metal coin cells. a) Specific discharge capacity and coulombic efficiencies with charge/discharge cycle number at 2C, 30 °C. Inset: schematic of LFP | in situ PTMC | Li coin cell setup. b) Rate performance of LFP | in situ PTMC | Li cell. c) Corresponding charge/discharge profiles for indicated cycle numbers.

Having established the thermal, mechanical, and electrochemical properties of the in situ PTMC electrolyte, the next step was to evaluate its long‐term cell cycling performance. These experiments were conducted using a commercial LiFePO_4_ (LFP) cathode and Li metal anode, with the electrolyte, at 30 °C (**Figure** **4**). In the literature, the cycling performance of other LFP | polymer electrolyte | Li metal batteries tends to be reported at low C‐rates (< 2C), and at high temperatures (>30 °C); there are very few reports where batteries are tested under more realistic or demanding operating conditions, i.e., fast C‐rates and at low/room temperature.^[^ ^19^, ^22^, ^31^ ^]^ Thus, we deliberately selected these challenging conditions, under which the PTMC semi‐solid electrolyte showed excellent long‐term capacity retention of 97% over 350 cycles at a high current rate of 2C (Figure 4a). The voltage curves showed very small changes in polarization (80 mV at 0.1C over 20 cycles), further supporting the high cycling stability (Figure 4c, Figure S13, Supporting Information). Furthermore, the cells using the in situ PTMC electrolyte showed good rate performances as exemplified by the high discharge capacity of 86 mAh g^−1^ obtained even at a high current density of 680 mA g^−1^ and 4C. When the cell was returned to slower charge rates (0.1C), it achieved nearly quantitative theoretical capacity retention (98.8% of theoretical, 168 mAh g^−1^) (Figure 4b). This exceptional performance at high rates can be attributed to the high ionic conductivity of the in situ PTMC electrolyte (0.52 mS cm^−1^), improved interfacial contact resulting from the in situ polymerization approach, and the use of LiDFOB as the salt, which is known to form favorable passivating SEI layers.

To gain further insight into the effects of the in situ polycarbonate electrolyte on cell performance, the morphology and composition of the interfacial region between in situ PTMC and lithium metal was characterised through electrochemical (EIS), microscopy (FIB‐SEM) and spectroscopic techniques (XPS, EDX) (Figures S15–S18, Supporting Information). EIS measurements carried out using symmetric Li cells showed a steady decrease in resistance within the first 10 h and plateauing after 50 h (Figure S16, Supporting Information), suggesting that the in situ PTMC electrolyte effectively maintains interfacial contact with Li metal forms a SEI that promotes stable lithium plating and stripping. Furthermore, FIB‐SEM revealed the presence of a homogeneous and smooth surface layer, supporting the idea that the in situ polymerisation leads to intimate contact with the electrode (Figure S17, Supporting Information). Finally, comparative galvanostatic cycling in an equivalent cell was performed using the liquid TMC monomer electrolyte, also with 10 mol% LiDFOB (Figure S14, Supporting Information). Both the semi‐solid polymer and cyclic carbonate electrolytes demonstrated comparable discharge capacities of 138 mAh g^−1^ at a rate of 1C (Figure 4b). However, the liquid cyclic carbonate, TMC, electrolyte exhibited a significantly faster capacity fade, retaining only 55% of its initial capacity after 500 cycles (Figure S15, Supporting Information). These findings are consistent with the rheological/mechanical and stability benefits afforded by using a semi‐solid polycarbonate electrolyte.

### 2.5 Recyclable and High‐Performance Polymer Electrolyte

**Figure 5:**
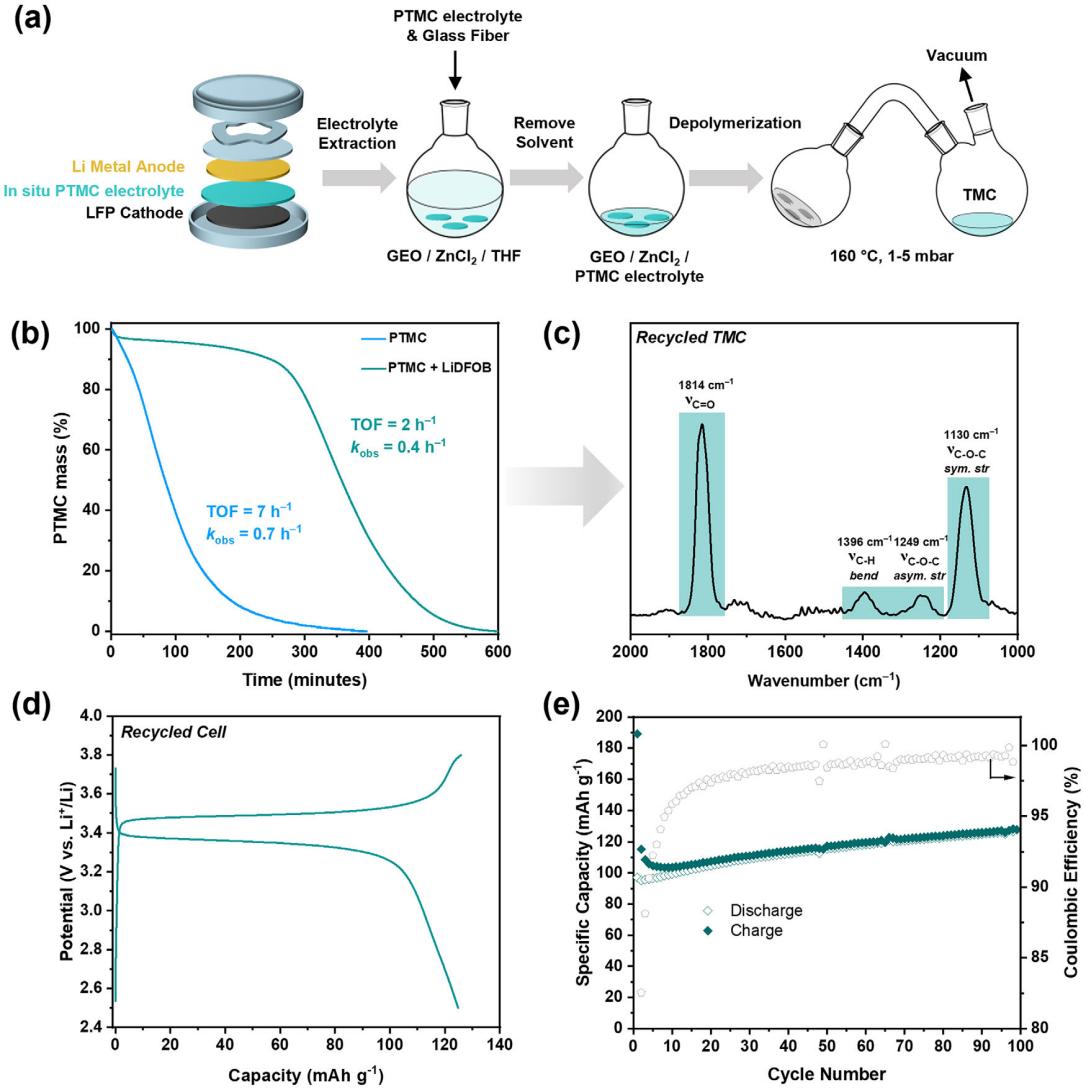
Chemical recycling of PTMC electrolyte and reuse in cells. a) Schematic flowchart of solid‐state recycling PTMC electrolyte from spent coin cells. b) TGA traces for small‐scale solid‐state chemical recycling of PTMC electrolyte with and without salt removal using ZnCl_2_ and GEO. c) FT‐IR spectrum of TMC monomer recovered during recycling. d) Charge/discharge profiles of a coin cell assembled with recycled PTMC electrolyte at 1C. e) Galvanostatic cycling performance and Coulombic efficiency of recycled PTMC electrolyte coin cell at 1C.

To achieve a circular economy for next‐generation rechargeable batteries, the removal of the polymer electrolyte may help recover the valuable inorganic metals and electrode materials. To test the recyclability of the PTMC electrolyte, we developed a small‐scale testing procedure by mixing the polymer electrolyte with a catalyst (ZnCl_2_) and non‐volatile alcohol (glycerol ethoxylate, GEO) in the solid state. Films of this mixture were subjected to isothermal thermogravimetric analysis (TGA); the conditions were selected since related protocols had previously proved very effective in monitoring related oxygenated polymer recycling.^[^ ^32^ ^]^ In the procedure, the polymer electrolyte (PTMC and LiDFOB) was mixed with the catalyst system (ZnCl_2_/GEO) in THF, and a sample of the mixture was added to a TGA crucible. The sample was dried under vacuum to remove all of the THF (which was only present a small scale to ensure good mixing of the polymer electrolyte and catalyst). The resulting film was heated (isothermally) to 160 °C in the TGA instrument, under a nitrogen flow of 25 mL min^−1^, and mass loss was monitored for 400–600 min (**Figure** **5**c). The PTMC mass loss vs. time data enabled the determination of depolymerization kinetics, following established procedures.^[^ ^32^ ^]^ The TGA instrument is equipped with an inline FT‐IR spectrometer, enabling analysis of the depolymerization products (Figure 5). TMC formation was confirmed by the IR spectroscopy showing its carbonyl (C═O) vibration at 1814 cm^−1^, C─H bending vibration at 1396 cm^−1^, and the C─O─C asymmetric and symmetric stretching vibrations at 1249 and 1130 cm^−1^, all at the same frequency as pure monomer samples. The data showed that the PTMC electrolyte was completely depolymerized in 10 h and that the only product formed was the TMC monomer. These findings are fully consistent with the expected depolymerization equilibrium proposed at the outset of the research (Figure 5b). The depolymerization of PTMC (i.e., no lithium salt) was considerably faster under equivalent conditions (reaching complete conversion to TMC in ≈6 h, Figure 5b). The slower rates suggest that the salt may stabilize the polymer alcohol chain end groups, perhaps through competitive coordination chemistry to Li.

Given the promising results obtained through small‐scale experiments, the solid‐state recycling was conducted at a larger‐scale using 0.8 g of in situ PTMC electrolyte. The polymer was isolated from cycled coin cells, which were disassembled in an argon‐filled glovebox. The polycarbonate electrolyte was extracted from glass fibre separators by soaking in the catalyst solution (ZnCl_2_/GEO in THF). In these experiments, the same loading of catalyst, alcohol, and polymer was used as in the small‐scale tests (GEO:ZnCl_2_:PTMC = 1:3:20, Figure 5a, Figure S21, Supporting Information). The solvent was removed under vacuum, and the resulting film was subjected to depolymerization, using short path distillation apparatus, at 160 °C and 1–5 mbar vacuum. Under these conditions, the recycling selectively formed pure TMC monomer, which was isolated in 62% yield after 24 h. The recovered TMC monomer was next reused to make the polymer electrolyte, using an identical in situ polymerization procedure (but with fresh Li‐salt and catalyst). The in situ, recycled PTMC electrolyte was produced in an identical cell configuration featuring an LFP cathode and Li anode. The recycled cell showed charge/discharge voltage profiles with minimal polarization (Figure 5d). The discharge capacity was 123 mAh g^−1^_,_ at a rate of 1C (Figure 5e), which is closely comparable to the performance of the original battery (125 mAh g^−1^, Figure 4b). Additionally, the recycled polycarbonate electrolyte displayed good cycling stability and a high Coulombic efficiency of ≈99.97% (Figure 5e). These findings demonstrate the potential to exploit the heterocycle ROP equilibrium to enable efficient and low energy (160 °C) polymer electrolyte recycling.

## 3 Discussion

Since the ability to use polymer electrolytes depends upon several factors, it is important that the in situ PTMC reported in this work is contextualised against the leading materials reported in the literature. First, the in situ PTMC electrolyte clearly shows an ionic conductivity at room temperature of 0.52 mS cm^−1^ which is higher than almost all other reported semi‐solid polymer electrolytes with the exceptions of a report of in situ PDOL using LiTFSI as the salt, which achieved a slightly higher conductivity of 1 mS cm^−1^_,_ and an excellent recent publication from Wang and co‐workers describing an in situ PTMC electrolyte using LiTFSI which exhibited a high ionic conductivity of 1.62 mS cm^−1^ at room temperature^[^ ^20^ ^]^ (Figure S23 and Table S2, Supporting Information). Polymer electrolytes that utilise LiTFSI as the source of Li^+^ ions typically demonstrate higher ionic conductivity due to the greater dissociation of cation–anion pairs. More broadly, it has been found that polyethers often show slightly higher conductivity than polycarbonates but there are, of course, other benefits afforded by the carbonate repeat unit (including low‐energy recycling).^[^ ^9b^ ^]^ There is significant prior literature focused on electrolytes comprising a large quantity of solvent, often formed in situ, and these are classed as gel polymer electrolytes (GPEs). In these GPEs, the Li‐ion transport typically occurs within the entrapped liquid electrolyte rather than by the polymer chains and this increases the conductivity of the in situ GPEs (Figure S23, Supporting Information). One drawback for gel polymer electrolytes is the presence of significant quantities of volatile and flammable solvents, e.g., ethylene carbonate, or small‐molecule additives such as fluoroethylene carbonate (FEC) or LiNO_3_. Thus, this work is more appropriately benchmarked against other polymer semi‐solid electrolytes. The in situ PTMC electrolyte shows a good high temperature stability, with the onset of decomposition occurring above 160 °C whereas the leading PDOL‐based electrolytes begin to decompose from 70–120 °C.^[^ ^19^, ^33^ ^]^ These properties for semi‐solid PDOL electrolytes may limit the battery operation temperatures compared to the polycarbonates reported here. The oxidative stability of the in situ PTMC electrolyte is 4.7 V vs. Li/Li^+^ which surpasses the values for poly(ethylene oxide)‐based electrolytes (> 4 V)^[^ ^34^ ^]^ and aligns with other polycarbonate‐based and PDOL containing electrolytes (*≈*4.4–5.1 V).^[^ ^19^, ^20^, ^30^, ^33^, ^35^ ^]^

The excellent battery performance of the in situ PTMC electrolyte is clear when comparing it to the literature battery cycling data. In conducting the analysis, we compared the specific discharge capacities of a series of leading in situ semi‐solid polymer electrolytes, in LFP | polymer electrolyte | Li cells, as a function of C‐rate (Figure S26 and Table S3, Supporting Information). The in situ PTMC electrolyte, in this work, displays the highest discharge capacity reported for any of the samples at room temperature, achieving a capacity of 170 mAh g^−1^ at 0.1C. It outperforms in situ PDOL electrolytes in terms of specific discharge capacity at 0.2 to 1C, under these conditions, values are 95–115 mAh g^−1^ for PDOL and 129–170 mAh g^−1^ for in situ PTMC. It also shows superior rate capability performance with charge‐discharge cycling being highly effective at 2–4C, in comparison the maximum C‐rate at which PDOL was cycled was 1C. This result is surprising given the high ionic conductivity (1 mS cm^−1^) and high oxidative stability (4.7 V vs. Li/Li^+^) of in situ PDOL, and can be ascribed to its instability against Li metal. The addition of LiDFOB, in dual salt systems, improves in situ PDOL significantly (Figure S26, Supporting Information). While this is an interesting approach, it significantly lowers the ionic conductivity compared to single‐salt in situ PDOL, even after the addition of succinonitrile (a plasticizer), reaching 0.1–0.2 mS cm^−1^ compared to 1 mS cm^−1^ for single salt PDOL. While LiDFOB is known to improve SEI stability in Li metal,^[^ ^27^ ^]^ the fact that superior battery performance is obtained here without the need for additives or dual‐salt electrolytes facilitates the recyclability of the electrolyte. The in situ polycarbonate (PTMC) electrolyte outperforms cross‐linked PEG networks which achieved 140 mAh g^−1^ at 0.05C and only modest rate performance up to 1C. These cross‐linked samples show lower ionic conductivity (0.03 mS cm^−1^) and would be challenging to recycle. A recent report also exploring in situ fabricated PTMC electrolytes showed comparable performance to this work with an initial capacity of 143 mA h g^−1^ at 0.2C. Whilst further testing at other rates was not performed in LFP cells, the authors demonstrate excellent compatibility in cells that employ high voltage cathode material NMC811, which exhibited an initial discharge capacity of 205 mA h g^−1^ at 0.1C and rate capability up to 2C.^[^ ^20^ ^]^

The long‐term capacity retention of the in situ PTMC was contextualized against the leading samples reported in the literature (Figures S24 and S25, Supporting Information). In these comparisons, the cell capacity retention after 100 cycles was evaluated since longer cycling was not available for some of the in situ semi‐solid electrolytes (Figure S24, Supporting Information). The PTMC electrolytes showed no significant degradation over this cycle length. The PEG‐based electrolytes also showed good stability but were generally cycled at much slower rate (0.1 vs. 2C for PTMC). Generally, in situ PDOL electrolytes showed lower capacity retention (90–50%) compared to the in situ PTMC reported in this work. This finding may arise from the lower oxidative stability of polyethers compared to polycarbonates.^[^ ^9a^ ^]^ Previous work has shown that the addition of succinonitrile and LiDFOB in PDOL further reduced the long‐term capacity retention, which was a function of rate (lower capacity retention at higher rates) suggesting that the use of this additive/salt combination might trigger additional degradation pathways. Where available, longer term cycling data was compared with other systems (Figure S25, Supporting Information). These comparisons reveal that the in situ PTMC electrolyte shows high‐capacity retention and long‐term cycling stability (97% after 350 cycles) compared to PDOL‐based in situ electrolytes (86–87%) even at higher C‐rates.

This report demonstrates how monomer/polymer equilibria can be exploited to facilitate the chemical recycling to monomer of a polymer electrolyte showing that the PTMC electrolyte can be efficiently recycled, at just 160 °C in the presence of a catalyst, to selectively recover the TMC monomer in good yield (62%). Recent work by Wang et al. has also demonstrated the closed‐loop chemical recycling of a in situ formed PTMC electrolyte using LiTFSI as both the polymerization and depolymerization catalyst.^[^ ^20^ ^]^ The recovery of both the TMC monomer (>90%) and LiTFSI (98%) was achievable in high yields without the addition of an external catalyst, albeit at greater temperatures of 180 °C. At the outset, our research targeted methods to make the polymer electrolyte in situ in the battery, which was effectively demonstrated. A key requirement was to ensure that the depolymerization was not triggered by any temperature spikes or higher temperature battery operation requirements. Our investigations reveal a beneficial catalyst and polymer chain end reactivity quenching by a small quantity of lithium salt. This interaction stabilizes the PTMC electrolyte enabling it to operate effectively over a wider temperature range. However, after use, the PTMC electrolyte, containing the lithium salt, can be effectively recycled to the TMC monomer indicating that the interactions do not interfere with the future recycling. The proof‐of‐concept monomer‐polymer equilibrium and recycling demonstrated in this work is significant since there is a very wide range of other heterocycle monomers which would be expected to behave similarly. Indeed, many 6‐ and 7‐membered ring cyclic carbonates and esters are known to exhibit related monomer‐polymer equilibria.^[^ ^36^ ^]^ Furthermore, those monomers are oxygenated and can be modified to include substituents which may further benefit conductivity, stability and interfacial properties. Thus, in future a series of other oxygenated polymers, including some polyethers,^[^ ^37^ ^]^ should be tested as both in situ electrolytes and their recyclability after use should be established. The future circular economy for batteries will require many advances, including effective methods to recover and reuse the metals and ceramics. The ability to recycle, and reuse, the polymer electrolyte is a first‐step to new recycling technologies.

## 4 Conclusion

A new route to make a high‐performance and recyclable poly(trimethylene carbonate) (PTMC) electrolyte is reported. The in‐cell fabricated polycarbonate electrolyte exhibited high ionic conductivity (0.52 mS cm^−1^ at 30 °C), and excellent oxidative stability (4.7 V vs. Li/Li^+^). Batteries configured with lithium metal and lithium iron phosphate (LFP), using the new in situ formed polycarbonate electrolyte, demonstrated outstanding capacity of 168 mAh g^−1^ at 0.1C, corresponding to 98.8% of theoretical capacity, impressive rate performance of 86 mAh g^−1^ at 4C, and remarkable cycling stability (97% capacity retention after 350 cycles at 2C). After use, the new polycarbonate electrolyte was recovered from the battery and efficiently recycled to its cyclic carbonate monomer (TMC). This recycling was conducted at 160 °C, using a catalysed, solid‐state process, and enabled isolation of pure monomer, which was repolymerized, in a new battery, to deliver equivalent cell cycling performance to the original cell. The recycling is enabled by exploiting the monomer‐polymer equilibrium which is present in many different classes of oxygenated polymer. This work demonstrates how polymerization thermodynamics can be used to ensure closed‐loop polymer electrolyte recycling. The concepts exemplified in this work apply to a wide range of other cyclic monomers including those featuring carbonate, ether and ester linkages, as well as with various substituents. Future explorations of these monomer‐polymer equilibria, using other chemistries, provide an exciting opportunity to tailor polymer manufacturing, properties and recycling to battery structures and chemistries.

## Conflict of Interest

The authors declare no conflict of interest.

## Supporting information



Supporting Information

## Data Availability

The data that support the findings of this study are available from the corresponding author upon reasonable request.
