# Supplementary material for: Recyclable Li‐Metal Battery Electrolytes via In Situ Cyclic Carbonate Polymerization
Source: Adv Sci (Weinh). 2025 Jun 9;12(32):e04206. doi: 10.1002/advs.202504206 (PMC12407366; doi:10.1002/advs.202504206)
Supplement: Supplementary file 1 — Supporting Information [file ADVS-12-e04206-s001.docx]

Supporting Information

**Recyclable Li-Metal Battery Electrolytes Via In Situ Cyclic Carbonate Polymerization**

*Hui Gao, Victor Riesgo-Gonzalez, James R. Runge, Kanyapat Yiamsawat, Dominic Spencer-Jolly, Thomas M. McGuire, Gregory J. Rees, Xiangwen Gao, Bingkun Hu, Shengming Zhang, Longlong Wang, Peter G. Bruce,* Georgina L. Gregory,* and Charlotte K. Williams**

H. Gao, V. Riesgo-Gonzalez, J. R. Runge, K. Yiamsawat, T. M. McGuire, G. L. Gregory, C. K. Williams

Chemistry Research Laboratory, University of Oxford, Oxford, OX1 3TA, UK.

E-mail: [georgina.gregory@chem.ox.ac.uk](mailto:georgina.gregory@chem.ox.ac.uk); [charlotte.williams@chem.ox.ac.uk](mailto:charlotte.williams@chem.ox.ac.uk)

H. Gao, D. Spencer-Jolly, G. J. Rees, X. Gao, B. Hu, S. Zhang, L. Wang, P. G. Bruce

Department of Materials, University of Oxford, Oxford, OX1 3PH, UK.

E-mail: [peter.bruce@materials.ox.ac.uk](mailto:peter.bruce@materials.ox.ac.uk)

H. Gao, V. Riesgo-Gonzalez, J. R. Runge, D. Spencer-Jolly, G. J. Rees

The Faraday Institution, Quad One, Harwell Science and Innovation Campus, Didcot, OX11 0RA, UK

Keywords: recycling, batteries, polymer electrolyte, lithium metal, in-situ polymerization

**Table of Contents**

[1. Materials 3](#_Toc197788337)

[2. Methods 3](#_Toc197788338)

[2.1 Fourier-Transform Infrared Spectroscopy (FT-IR) 3](#_Toc197788339)

[2.2 Nuclear magnetic resonance (NMR) spectroscopy 3](#_Toc197788340)

[2.3 Differential Scanning Calorimetry (DSC) 3](#_Toc197788341)

[2.4 Thermal Gravimetric Analysis – Fourier-Transform Infrared Spectroscopy (TGA-FT-IR) 4](#_Toc197788342)

[2.5 Size Exclusion Chromatography (SEC) 4](#_Toc197788343)

[2.6 Rheology 4](#_Toc197788344)

[2.7 Powder X-ray diffraction (PXRD) 4](#_Toc197788345)

[2.8 X-ray Photoelectron Spectroscopy (XPS) 5](#_Toc197788346)

[2.9 Focussed Ion Beam Scanning Electron Microscopy with Energy-dispersive X-ray Spectroscopy (FIB-SEM/EDX) 5](#_Toc197788347)

[2.10 Linear Sweep Voltammetry (LSV) 5](#_Toc197788348)

[2.11 Electrochemical Impedance Spectroscopy (EIS) 5](#_Toc197788349)

[2.13 Galvanostatic Discharge-charge 6](#_Toc197788350)

[3. Experimental Procedures 6](#_Toc197788351)

[3.1 Preparation of in situ PTMC electrolyte 6](#_Toc197788352)

[3.2. Preparation of PTMC for TGA depolymerization experiments 7](#_Toc197788353)

[3.3 Preparation of Electrodes 7](#_Toc197788354)

[3.4 Fabrication of Coin cells 7](#_Toc197788355)

[3.5 TMC/PTMC equilibrium 7](#_Toc197788356)

[3.6. Recycling of PTMC electrolyte 8](#_Toc197788357)

[4. ROP mechanism 9](#_Toc197788358)

[5. Characterization Data of PTMC Electrolyte 9](#_Toc197788359)

[NMR spectroscopy 9](#_Toc197788360)

[Size Exclusion Chromatography (SEC) 13](#_Toc197788361)

[Thermal Characterisation 15](#_Toc197788362)

[Powder X-ray Diffraction 16](#_Toc197788363)

[Monomer/Polymer Equilibrium Data 16](#_Toc197788364)

[Electrochemical Characterisation 17](#_Toc197788365)

[5. Cell Performance 19](#_Toc197788366)

[6. Surface characterisation of cycled lithium anode 21](#_Toc197788367)

[7. Solid-state Recycling 23](#_Toc197788368)

[8. Comparison to Literature 25](#_Toc197788369)

[9. References 36](#_Toc197788370)

# 1. Materials

Trimethylene carbonate (TMC) was purchased from Tokyo Chemical Industry UK Ltd and purified by recrystallization from dry diethyl ether (× 3) and stored in a glovebox before use. Lithium difluoro(oxalato)borate (LiDFOB) was purchased from Sigma-Aldrich and dried under vacuum at 110 °C for 48 h before being stored in a glovebox. Phosphazene base P_2_-*t*Bu solution (2 M in THF) was purchased from Sigma-Aldrich and stored in a glovebox. Glass fiber separators were bought from Sigma-Aldrich and punched into discs (ф = 18 mm), dried under vacuum at 120 °C for 24 h before being stored in a glovebox. Lithium chips were purchased from Nanografi Nano Technology with a diameter of 16 mm and a thickness of 0.6 mm and stored in a glovebox. Lithium iron phosphate (LFP) was purchased from MSE Supplies. Polyvinylidene fluoride (PVDF) and *N*-methyl-2-pyrrolidone (NMP) were bought from Sigma-Aldrich. Carbon black (super P) was purchased from Alfa Aesar.

# 2. Methods

## 2.1 Fourier-Transform Infrared Spectroscopy (FT-IR)

FT-IR Spectra were obtained on a Shimadzu IRSpirit spectrometer, fitted with a KBr window and DLATGS detector with temperature control. FT-IR spectra were recorded inside a glove box using a single reflection ATR accessory and measured in transmission scanning mode. Solid polymer films were scanned from 4700-340 cm^–1^ (100 scans, 4 cm^–1^ resolution).

## **2.2 Nuclear magnetic resonance (NMR) spectroscopy**

^1^H NMR spectra were recorded on a Bruker Avance III HD 400 MHz spectrometer. Polymerization conversions were determined by ^1^H NMR spectroscopy.

## **2.3 Differential Scanning Calorimetry (DSC)**

Polymer and solid polymer electrolytes were analyzed on a DSC25 (TA Instruments) under a nitrogen flow (80 mL min^−1^). A sealed, empty crucible was used as a reference, and the DSC was calibrated using sapphire and indium. Samples were heated from -75 °C to 136 °C and held for 5 minutes to remove any thermal history before heating and cooling from -75 to 136 °C at a rate of 10 °C min^–1^. Glass transition temperatures (*T*_g_) were determined from the midpoint of the transition in the second heating curve.

## 2.4 Thermal Gravimetric Analysis – Fourier-Transform Infrared Spectroscopy (TGA-FT-IR)

Data was collected using a TGA5500 system (TA Instruments), equipped with a Nicolet iS20 FT-IR spectrometer (Thermo Scientific Instruments). The TGA temperature was calibrated against standards of alumel (Curie point = 153.0 °C), nickel (Curie point = 358.2 °C), nickel-83:cobalt-17 (Curie point = 554.4 °C), and nickel-63:cobalt-37 (Curie point = 746.4 °C). The TGA mass was calibrated against standards of 100.0000 mg and 1000.0000 mg. The FT-IR spectrometer was equipped with a KBr/Ge beamsplitter, fast-recovery deuterated triglycine sulfate KBr detector, and solid-state diode laser. FT-IR spectra were recorded between 400–4000 cm^–1^ with 10 scans per spectrum, at a resolution of 8 cm^–1^.

## 2.5 Size Exclusion Chromatography (SEC)

Polymer (2-10 mg) dissolved in HPLC grade CHCl_3_ (1 mL) was syringe filtered through 2 μm filters before being injected into an Agilent PL GPC-50 instrument, with two PSS SDV 5 μm linear M columns heated to 30 °C. HPLC grade CHCl_3_ was used as the eluent at a flow rate of 1.0 mL min^–1^ with RI detection calibrated using a series of narrow molecular weight polystyrene standards. Agilent size exclusion chromatography post-run program was used to analyze the data.

## 2.6 Rheology

Oscillatory shear rheology was conducted on a TA instruments Q800 with 25 mm stainless steel plates to monitor the polymerization reaction in real time. The oscillatory shear experiments were carried out with strain γ = 1% and angular frequency ω = 1 rad s^−1^ at 30 °C to 90 °C for 2 h. Reaction components were taken out of the glove box and mixed immediately prior to being loaded in the instrument.

## 2.7 Powder X-ray diffraction (PXRD)

PXRD patterns were obtained for the as-prepared materials using a Cu source Rigaku SmartLab diffractometer equipped with a Ge(220) double-bounce monochromator and a Hypix 2D detector. analysis system (GSAS) suite of programs. Ex situ PXRD was collected on a Cu source Rigaku Miniflex benchtop diffractometer housed within an N_2_-filled glovebox (O_2_ < 0.1 ppm, H_2_O < 0.1 ppm).

## 2.8 X-ray Photoelectron Spectroscopy (XPS)

Measurements were carried out using a PHI Versaprobe III XPS under ultrahigh vacuum, with a main chamber pressure of 10^-6^-10^-7^ Pa. The instrument uses an Al *Kα* monochromatic source and was operated at a power of 25 W and an electron beam voltage of 15 kV. A pass energy of 55 eV was set to acquire core-level spectra. Charge neutralisation was carried out with a low-energy BaO electron source. Samples were transferred into the instrument using an air-tight transfer module to prevent sample contamination. Data analysis was carried out using the CasaXPS software package. Spectra were fitted using a Shirley background and a Gaussian-Lorentzian lineshape. All spectra were charge referenced to adventitious carbon at 284.8 eV.

## 2.9 Focussed Ion Beam Scanning Electron Microscopy with Energy-dispersive X-ray Spectroscopy (FIB-SEM/EDX)

FIB-SEM was carried out using a Helios G4 PFIB CXe Dual Beam (Thermo Scientific). The sample was loaded into the instrument using an argon-filled transfer vessel (Gatan) and a cross section was milled with a Xe^+^ beam using a 30 kV accelerating voltage and a 4 nA current. SEM and EDX were carried out using a 10 kV accelerating voltage, with EDX maps acquired using an Oxford Instruments Ultim Max 170 X-ray detector.

## 2.10 Linear Sweep Voltammetry (LSV)

Recorded for in situ PTMC electrolytes on an MPG-200 series battery cycler (Biologic). Initial measurements were recorded at 30 °C vs lithium foil in a coin cell with a stainless steel (SS) counter-electrode (Li | PTMC | SS). The open-circuit voltage (OCV) was first recorded for 4 hours to ensure stability before conducting experiments at 0.1 mV s^–1^ from the OCV to 6.5 V.

## 2.11 Electrochemical Impedance Spectroscopy (EIS)

EIS was carried out on a Biologic impedance Analyzer from 30 to 80 °C with 0.5 h soak time at each temperature and between a frequency range from 0.1 MHz to 100 MHz, with an applied sinusoidal voltage amplitude of ±10 mV. The resulting Nyquist curves were analyzed using EC-labs software to fit an equivalence circuit and determine resistance, R. Conductivity (σ) was then determined as: σ = *l*/RS, where S is the polymer electrolyte disc area and *l* is the electrolyte thickness.

The temperature-dependent of ionic conductivity behavior for many polymer electrolytes can be described by the Vogel–Tammann–Fulcher (VTF) empirical equation:

$\sigma=AT^{-1/2}exp(\frac{{-E}_{a}}{R(T-T_{0})})$ (Equation S1)

where *A* is the conductivity pre-exponential factor, which is related to the number of carrier ions, *E*_a_ is the activation energy, *T*_0_ is the Vogel scaling temperature at which the free volume disappears or at which configuration free entropy becomes zero (generally taken to be *T*_g_ – 50 K), and R is the ideal gas constant.

**2.12. Symmetric Cell Cycling**

Symmetric galvanostatic cell cycling was recorded on an MPG-200 series battery cycler. A coin cell was assembled comprising of the in situ PTMC electrolyte and two lithium chips acting as the counter and reference electrodes (Li | PTMC | Li). For these experiments the areal capacity was set at 0.5 mA h cm^–2^. Cycling stability *vs.* Li metal was studied by cycling for 1000 h at a current density of 0.5 mA cm^–2^. The critical current density was confirmed by performing galvanostatic cycling from 0.1 to 2 mA cm^–2^ in steps of 0.1 mA cm^–2^ after each cycle.

## 2.13 Galvanostatic Discharge-charge

Cycling performance of the LFP│in situ PTMC electrolyte│Li coin cells was measured using a Maccor Series 4000 battery cycler. All potentials are given versus Li^+^/Li. All electrochemical measurements were performed on the coin cells at 30 °C. The cells were rested at 30 °C for 4 h before commencing the galvanostatic charge/discharge measurements.

# 3. Experimental Procedures

## 3.1 Preparation of in situ PTMC electrolyte

In an Ar-filled glovebox, TMC (100 mg, 0.97 mmol, 100 equiv.) and 10 mol% LiDFOB (14.1 mg, 0.097 mmol, 10 equiv., Li: C=O molar ratio of 10: 1) were mixed in a vial. To the resulting transparent liquid 6 mol % P_2_-*t*Bu catalyst vs. TMC was added as a 2M THF solution (29.39 µL, 0.059 mmol, 6 equiv.) for reactions conducted outside of the coin cell. For those performed in situ in the coin cell, 100 μL of TMC/LiDFOB mixture (131 mg) were injected onto a glass fiber separator followed by addition of 6 mol% vs. TMC P_2_-*t*Bu catalyst (2M THF solution). Coin cells were rested for 3h at 30 °C after assembling and before measurements were taken. Experiments conducted outside of the cell were not stirred to mimic the cell conditions. The progress of the reaction was monitored at different time intervals by quenching the base catalyst with one drop of acetic acid, adding 2 mL of CDCl_3_ and conducting ^1^H NMR analysis to assess the conversion of TMC monomer to PTMC polymer. For ionic conductivity measurements, the in situ PTMC electrolyte was formed on a glass fiber separator between two stainless steel blocking electrodes in a coin cell (Fig. 3a). Similarly, the reaction was conducted between one stainless steel and one lithium metal electrode for LSV measurements (Fig. 3b) and two lithium metal electrodes for lithium plating/stripping experiments (Fig. 3c).

## 3.2. Preparation of PTMC for TGA depolymerization experiments

PTMC (89.9 kg mol^-1^, *Đ* = 1.56) for TGA depolymerization experiments was synthesised according to literature procedure.^[1]^

## 3.3 Preparation of Electrodes

The positive electrodes were prepared using the following procedure: lithium iron phosphate (LFP) (350 mg, 70 wt%), carbon black (100 mg, 20 wt. %), and 10 wt.% PVDF (50 mg) in NMP solution with the mass ratio of 7: 2: 1 were charged into a vial equipped with a stirrer bar. Additional NMP was added to adjust the viscosity to form a slurry. After stirring the suspensions for 12 h, finely dispersed slurries were obtained, and these were then coated onto aluminium foil substrates (thickness × width × height = 0.005 × 16 × 25 cm) using a doctor blade at a fixed thickness (125 µm). The substrates were then dried at room temperature for 6 hours followed by drying under vacuum at 80 ℃ for 12 h to afford dry films. The films were then punched into discs with diameters of 12 mm. The discs were dried overnight under vacuum at 80 ℃ and transferred directly into a glovebox kept under an Ar atmosphere with H_2_O and O_2_ concentrations ≤ 0.1 ppm. The resulting electrode loadings were in the range of 1 mg cm^–2^.

## 3.4 Fabrication of Coin cells

Coin cells (CR2025) were assembled inside an Ar-filled glovebox. Li foil with a diameter of 16 mm and a thickness of 0.38 mm was used as the counter electrode, and glass microfiber (Whatman GF/F) was used as the separator. The separator was used to assemble cells to provide separation and control the thickness of the electrolyte. After preparation, the coin cells were taken out of the glovebox and rested for 3 h at 30 °C before testing.

## 3.5 TMC/PTMC equilibrium

The in situ reaction was modelled in vials without stirring to obtain conversion vs. time data from ^1^H NMR aliquots (Figure 1b). To do this, crystalline TMC (30.6 mg, 0.3 mmol) was combined with 10 mol% of lithium difluoro(oxalato)borate (LiDFOB) (4.3 mg, 0.03 mols), which lowered the melting point of the monomer to form a liquid precursor solution. Organocatalyst P_2_-*t*Bu (6 mol%, 2M in THF, 5.25 µL) was then added, and the reaction was conducted at 30 °C without stirring.

Additional experiments were carried out in acetonitrile (0.5 M in TMC) to determine the polymerization equilibrium thermodynamics (Δ*H*, Δ*S*)*.* Crystalline TMC (54 mg, 0.5 mmol) was dissolved in acetonitrile (1 mL). Organocatalyst 2M of P_2_-*t*Bu (0.5 mol%, 1.32 µL) was then added, and the reaction was conducted at 30 - 80 °C.

## 3.6. Recycling of PTMC electrolyte

**Recycle the polymer electrolyte by the solid-state method**

**Study the depolymerization using isothermal methods on the TGA coupled to an FTIR spectrometer:** PTMC (200 mg, 1.96 mmol, 20 equiv.), ZnCl_2_ (39 mg, 0.296 mmol, 3 equiv.) and glycerol ethoxylate (GEO, *M*_n_ = 1,000 g mol^–1^, 98 mg, 0.098 mmol, 1 equiv.) were added to a vial (molar ratio PTMC: ZnCl_2_: GEO = 20: 3:1). The mixture was dissolved in THF (1.96 mL) and 15 µL of the resulting mixture was drop-cast into a TGA crucible. Afterwards, the mixture was dried under vacuum for 30 minutes and exposed to an isotherm at 160 °C with a N_2_ flow rate of 25 mL min^–1^ for 300 minutes.

The depolymerization was then conducted on a larger scale using a short-path distillation setup (Fig. S19). Glass fibers (0.8 g) with polymer electrolyte (82% of the total mass) were placed into a reaction flask along with a ZnCl_2_/GEO solution (PTMC: ZnCl_2_: GEO = 20:3:1). The solvent was subsequently evaporated under vacuum, and the mixture was exposed to depolymerization conditions (160 °C, 1-5 mbar). After 24 hours, TMC was obtained with a yield of 62%.

# 4. ROP mechanism

**Scheme S1**. **Ring opening polymerization of TMC**. Structure of P_2_-*t*Bu catalyst is provided in Fig. S17 of the supporting information. Residual 1,3-propanediol present in TMC is assumed to act as the initiator.

# 5. Characterization Data of PTMC Electrolyte

## NMR spectroscopy


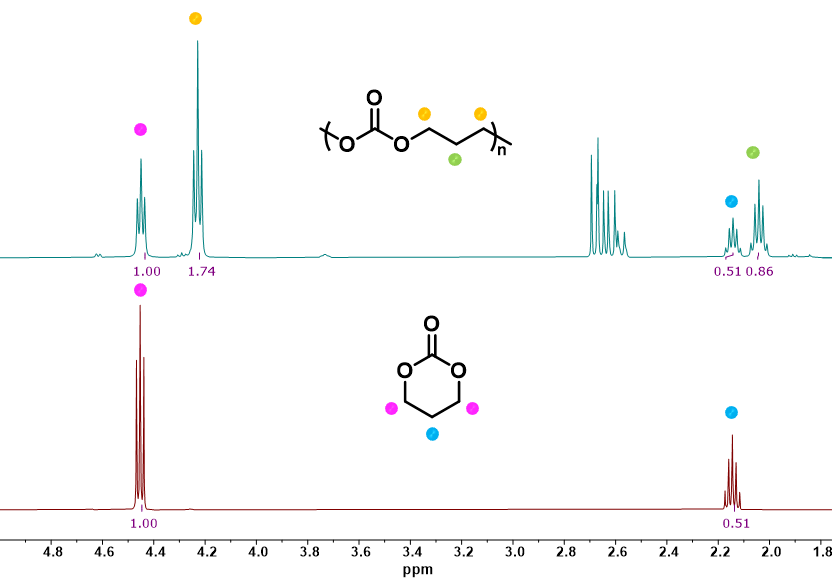


**Fig. S1.** ^1^H NMR spectra (400 MHz, CDCl_3_, 295K) of TMC and in situ PTMC. The reaction was carried out inside a coin cell (see experimental methods). Signals between 2.8-2.6 ppm correspond to the P_2_-*t*Bu catalyst.


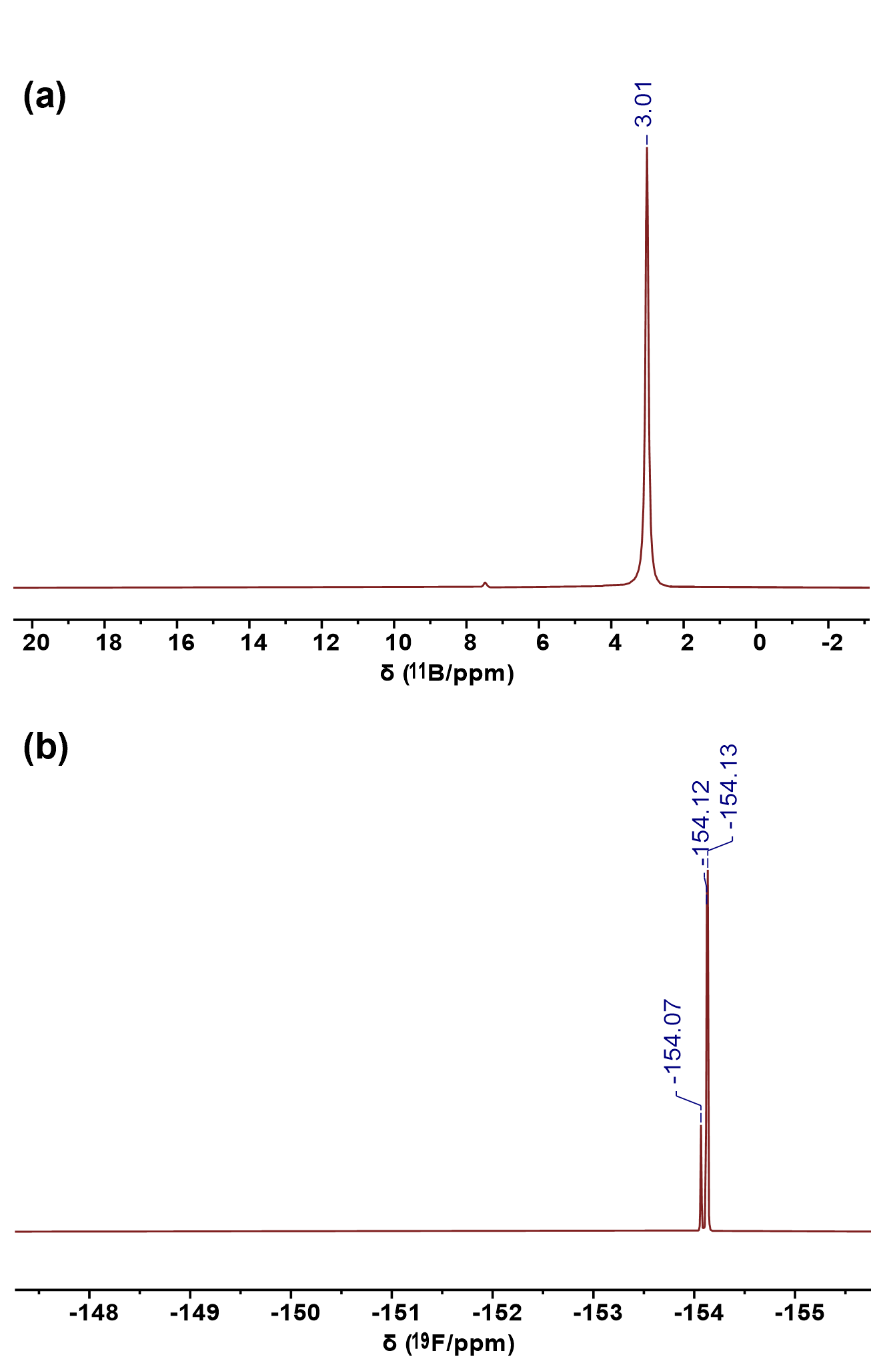


**Fig. S2:** (a) ^11^B NMR spectrum (128 MHz, CD_3_CN, 295 K) of lithium difluoro(oxalato)borate (LiDFOB). (b) ^19^F NMR spectrum (376 MHz, CD_3_CN, 295 K) of lithium difluoro(oxalate)borate (LiDFOB).


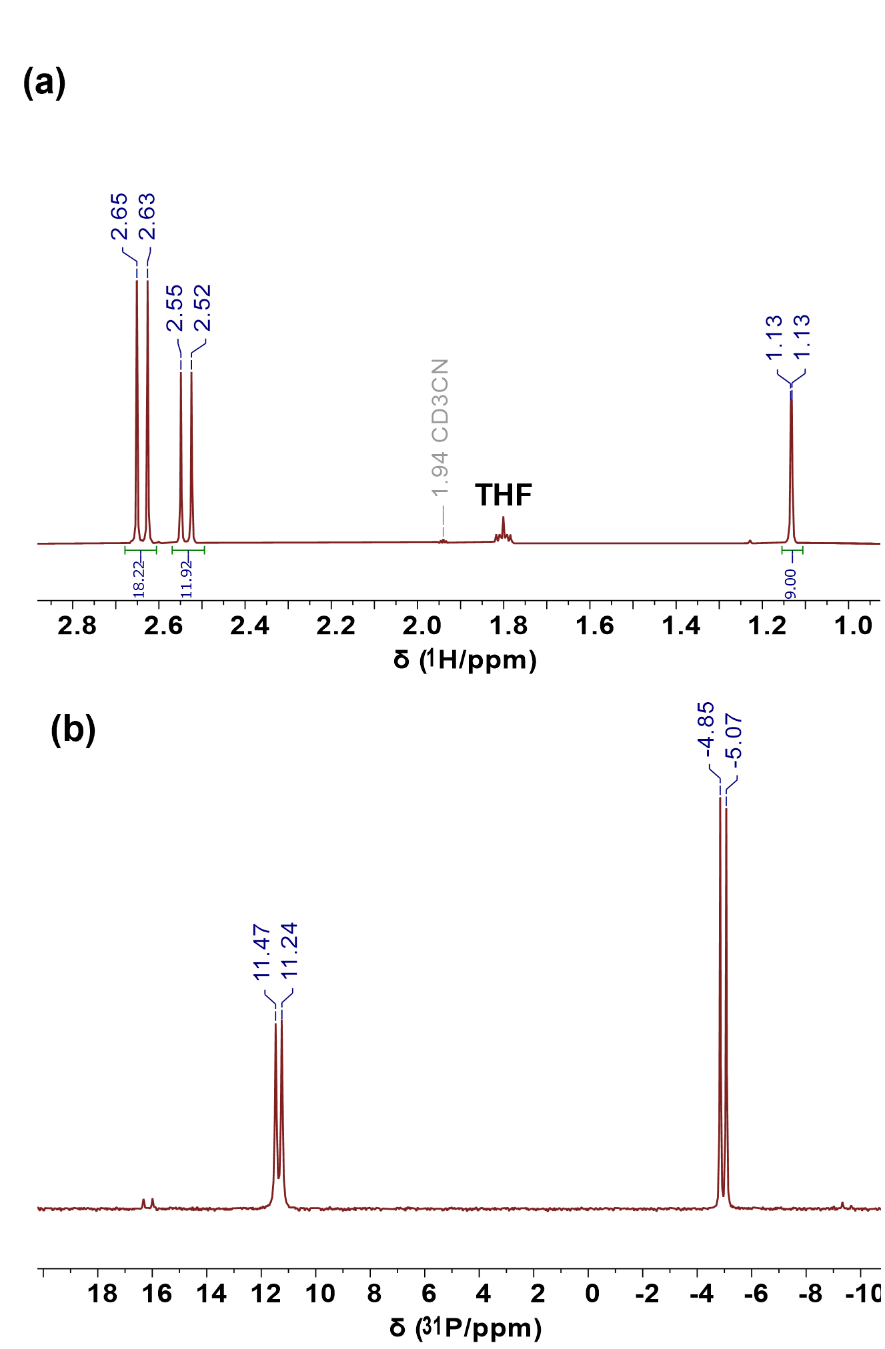


**Fig. S3**: (a) ^1^H NMR (400 MHz, CD_3_CN, 295 K) of P_2_-*t*Bu catalyst (2 M solution in THF) (b**)** ^31^P NMR (162 MHz, CD_3_CN, 295 K) of P_2_-*t*Bu catalyst (2 M solution in THF).


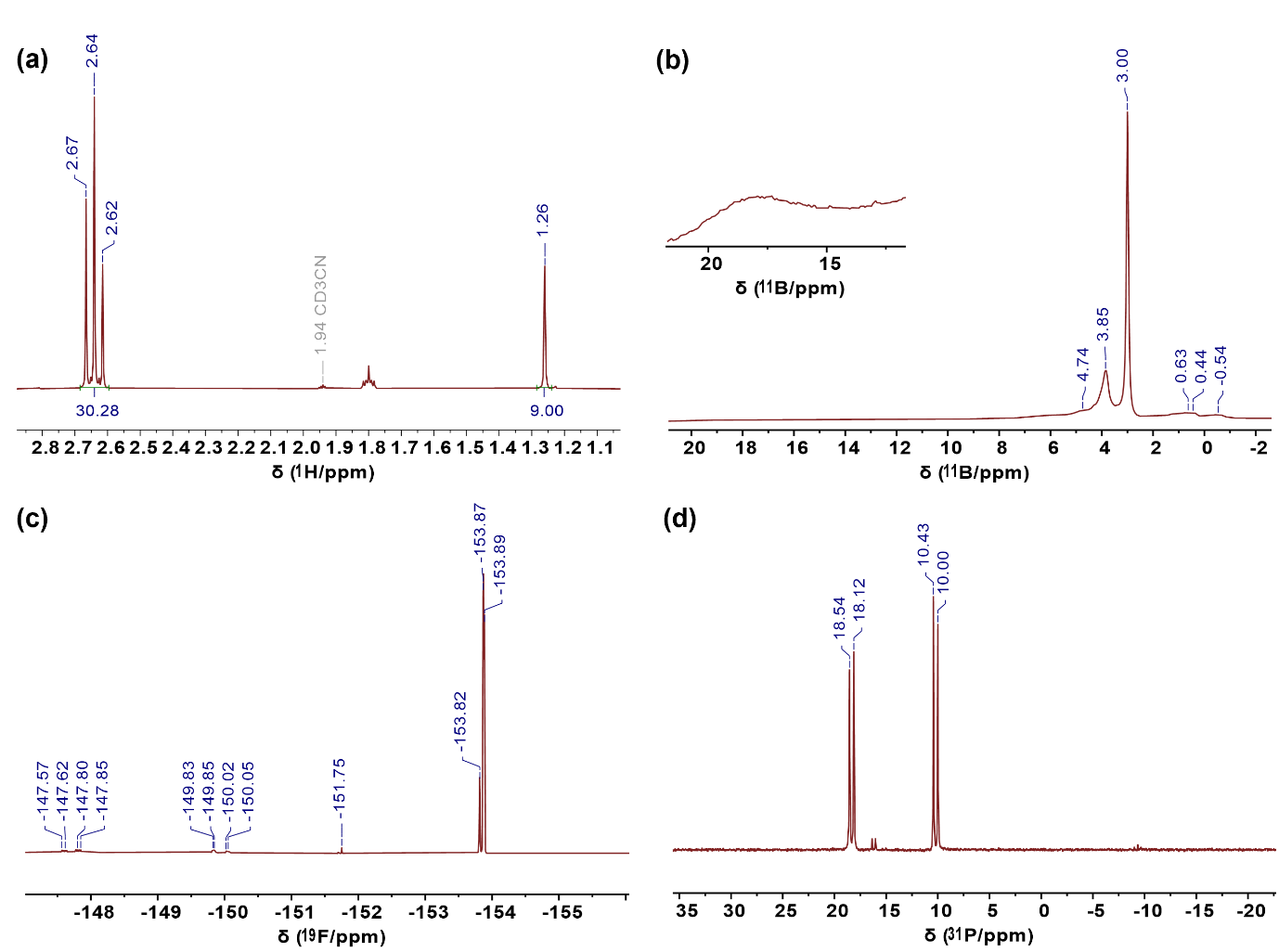


**Fig. S4:** (a) ^1^H NMR (400 MHz, CD_3_CN, 295 K) or reaction mixture of LiDFOB and P_2_-*t*Bu after 3 hours. (b) ^11^B NMR spectrum (128 MHz, CD_3_CN, 295 K) of reaction mixture of LiDFOB and P_2_-*t*Bu after 3 hours (c) ^19^F NMR spectrum (376 MHz, CD_3_CN, 295 K) reaction mixture of LiDFOB and P_2_-*t*Bu after 3 hours. (d) ^31^P NMR spectrum (162 MHz, CD_3_CN, 295 K) of reaction mixture of LiDFOB and P_2_-*t*Bu after 3 hours. Further characterization of the by-product was not undertaken however, these experiments clearly show indicate the formation of new species from the reaction of LiDFOB and P_2_-*t*Bu.

## Size Exclusion Chromatography (SEC)

**Fig. S5.** SEC trace of the in situ PTMC electrolyte measured in CHCl_3_ eluent against narrow polystyrene standards. Typically, dispersity values for PTMC conducted in solution and with stirring are < 1.5 owing to the ‘living’ controlled nature of the ring-opening polymerization mechanism. It is postulated that broad dispersity of the polymer is a result of inhibition of the polymerization by LiDFOB. This could lead to greater dispersity values as the polymer chain ends die during the polymerization process.

**Fig. S6.** SEC trace of the in situ PTMC electrolyte after cycling measured in CHCl_3_ eluent against narrow polystyrene standards.

## Thermal Characterisation

**Figure S7.** Additional TGA data of in situ PTMC electrolyte, pristine PTMC synthesised by P_2_-*t*Bu catalyzed ROP without addition of LiDFOB (i.e. no residual TMC present) and of unpolymerized TMC monomer.

## Powder X-ray Diffraction


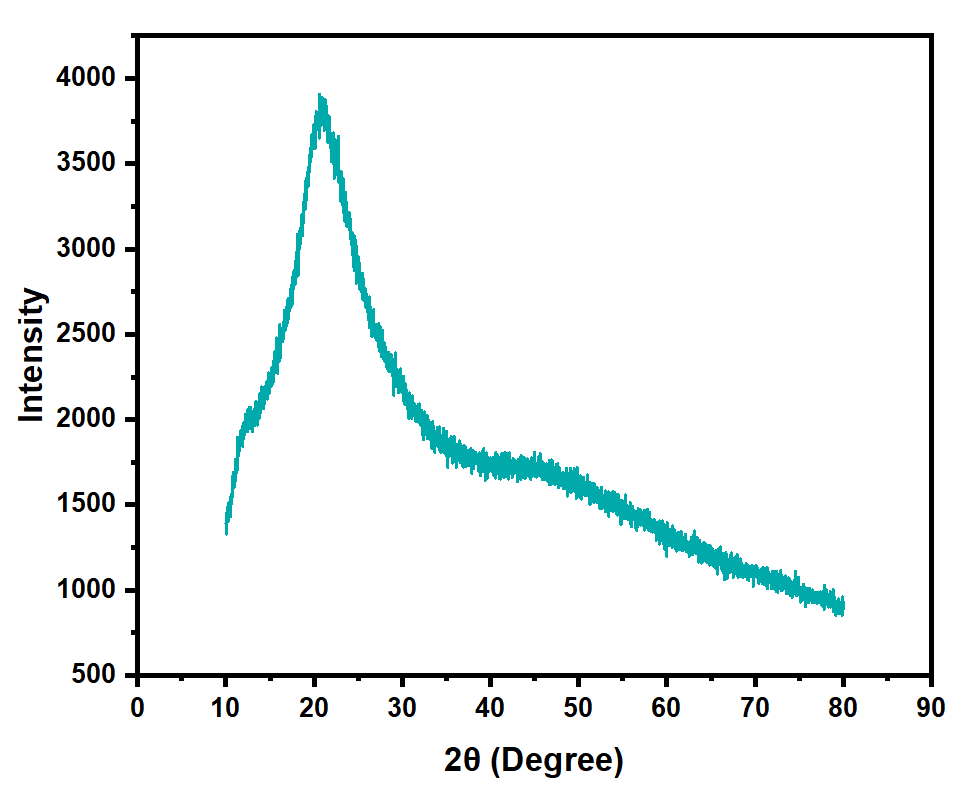


**Fig. S8.** PXRD pattern of the in situ PTMC electrolyte consistent with amorphous structure.

## Monomer/Polymer Equilibrium Data


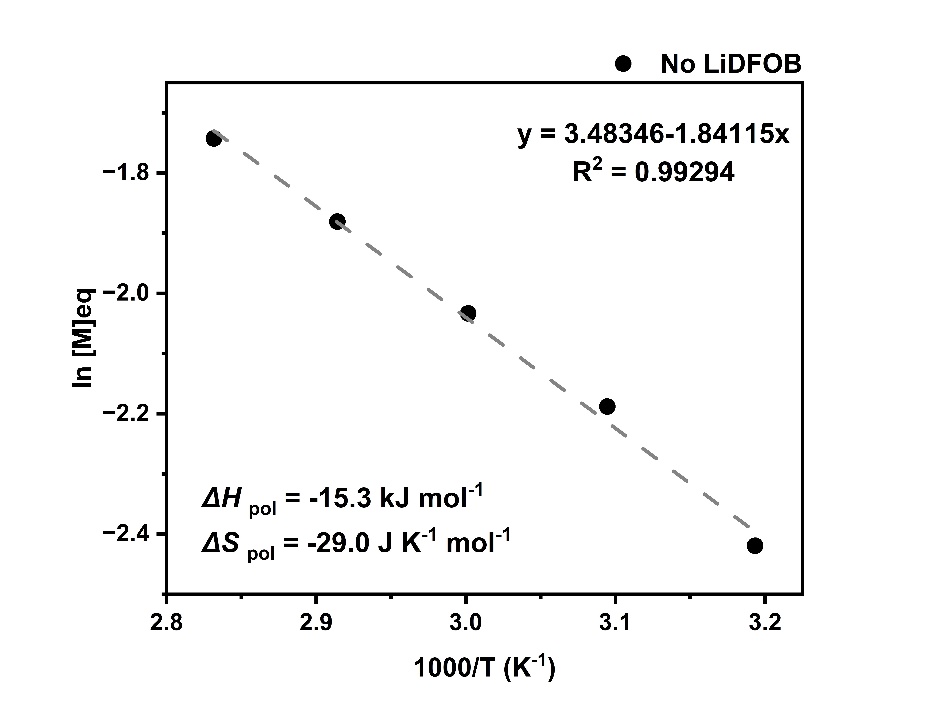


**Figure S9.** Polymerization thermodynamics of TMC (0.5 M in acetonitrile) in the presence of 6 mol% P_2_-*t*Bu.

## Electrochemical Characterisation


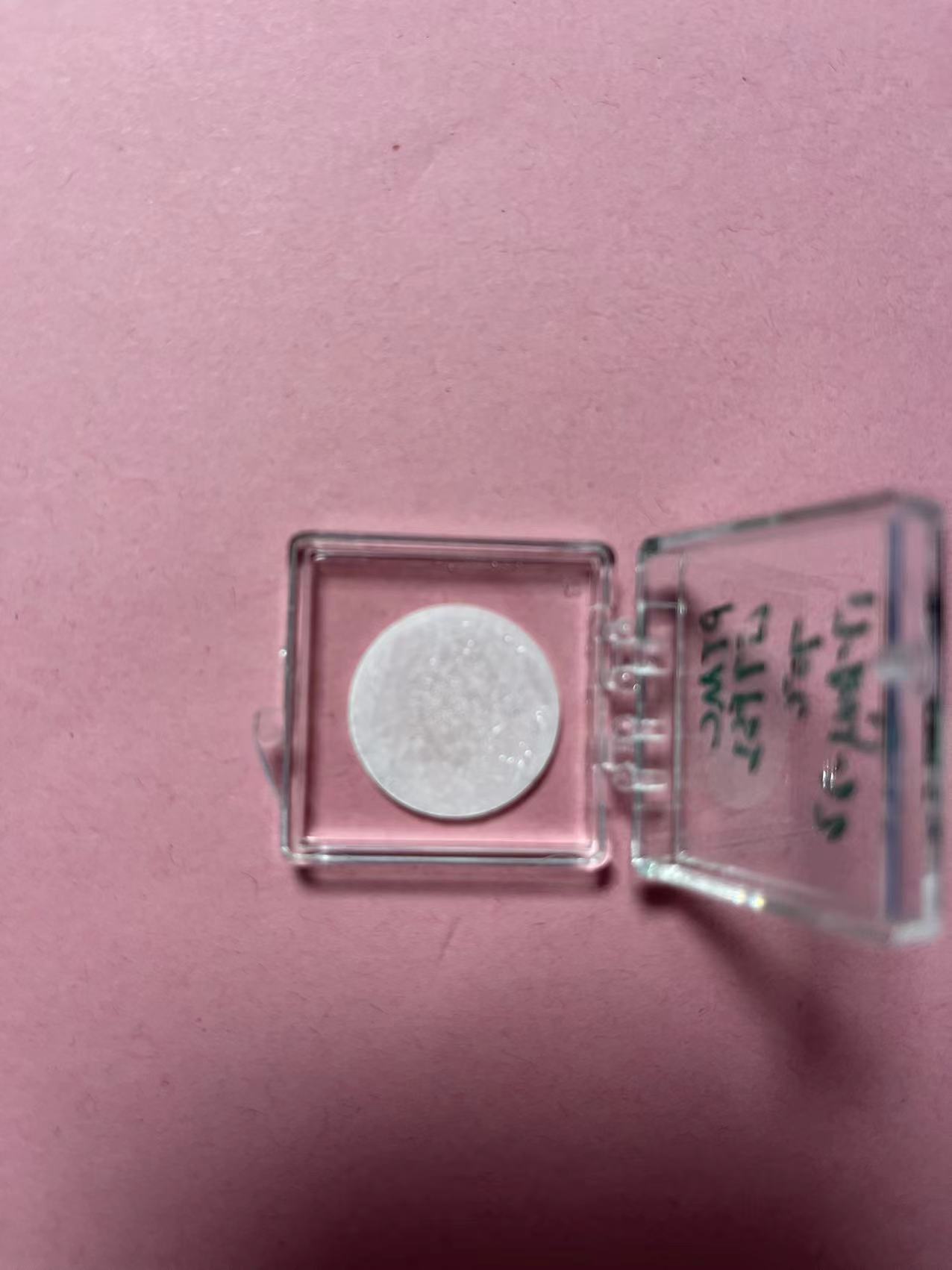


**Fig. S10**. Digital image of the in situ fabricated PTMC electrolyte on glass fiber.


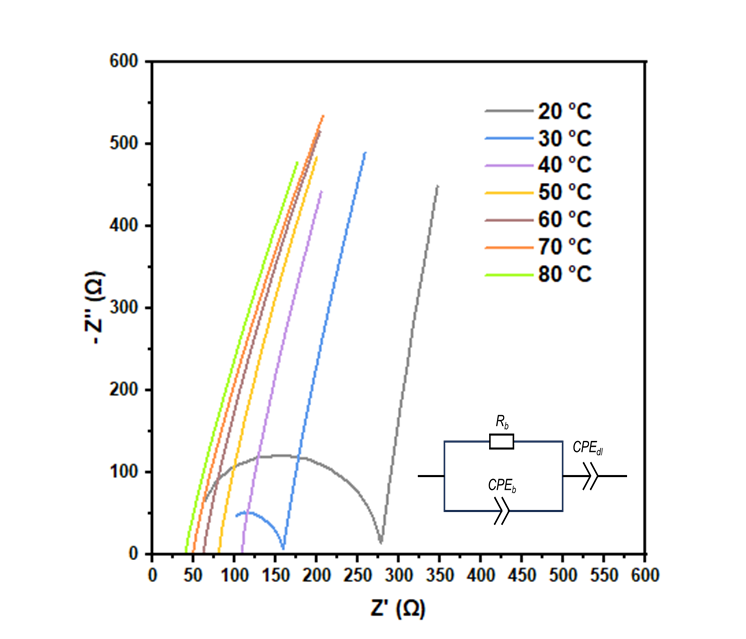


**Fig. S11.** Variable temperature electrochemical impedance spectra of *in situ* PTMC electrolyte with LiDFOB with the cell configuration of stainless steel │in situ PTMC electrolyte│stainless steel. The impedance spectra were fitted to an equivalent circuit (inset in the Figure) that consists of a constant-phase element (CPE_dl_) of the double layer in series with the parallel combination of a constant phase element (CPE_b_) of the bulk electrolyte and a resistance (*R*_b_) of the electrolyte.

**Table S1**. The ionic conductivity of in situ PTMC-LiDFOB as a function of temperature.

| **Temperature (^o^C)** | **In situ PTMC-LiDFOB** |
| --- | --- |
|  | **Ionic conductivity (S cm^–1^)** |
| 20 | 3.4x10^-4^ |
| 30 | 5.2×10^-4^ |
| 40 | 7.7×10^-4^ |
| 50 | 1.1×10^-3^ |
| 60 | 1.5×10^-3^ |
| 70 | 1.9×10^-3^ |
| 80 | 2.3×10^-3^ |


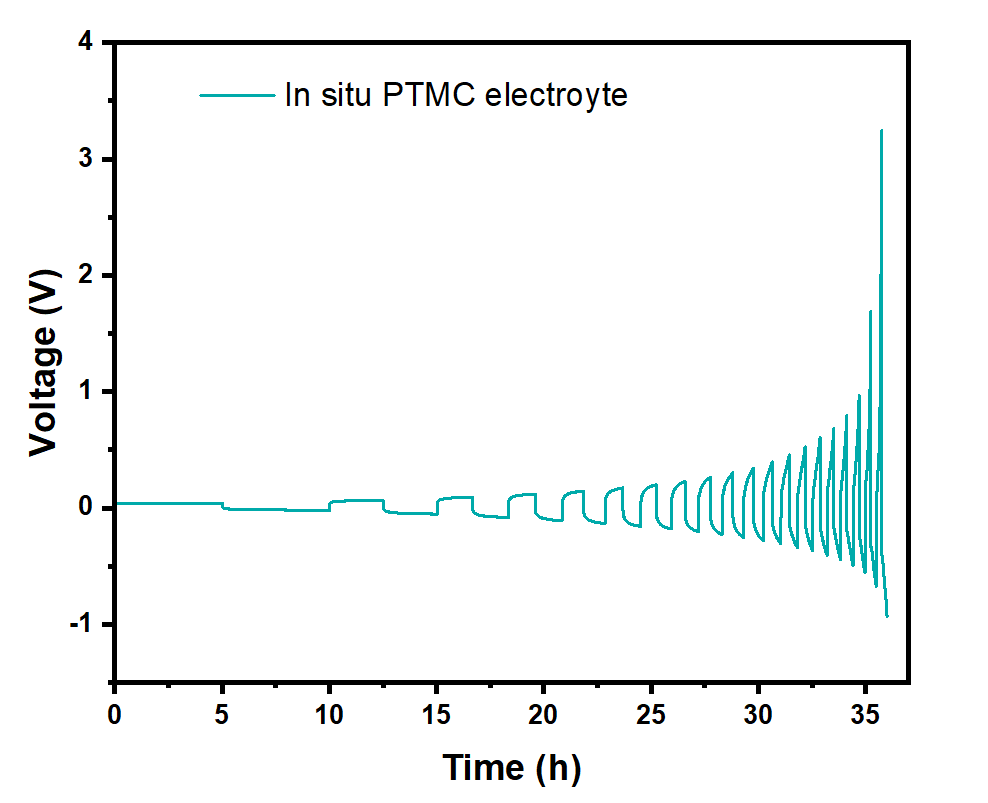


**Fig. S12**. Li stripping and plating profiles as a function of different current with an areal capacity of 0.5 mAh cm^–2^. The current density was increased in each cycle from 0.1 to 2 mAcm⁻² in steps of 0.1 mA cm⁻².

# 5. Cell Performance


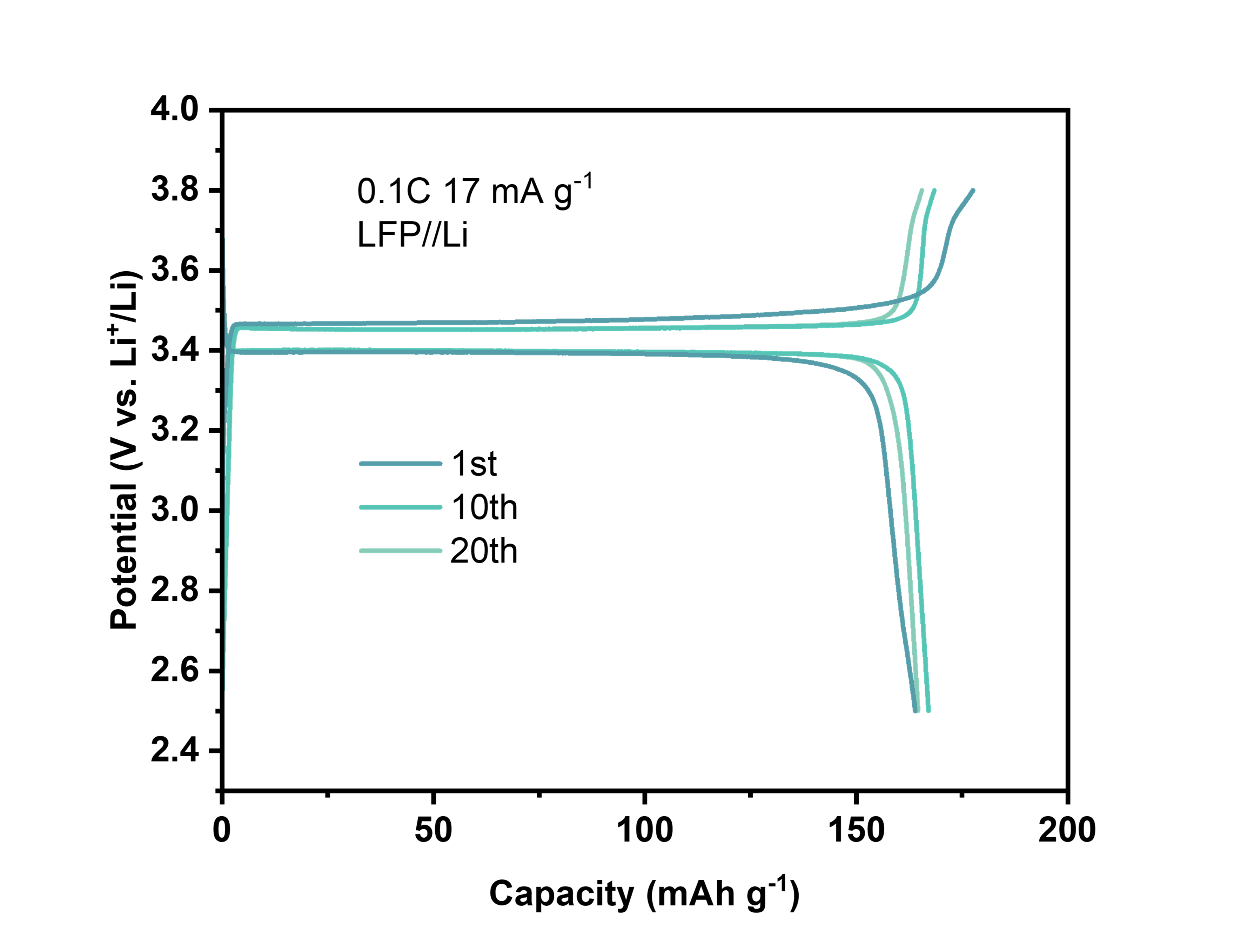


**Fig. S13**. Charge/discharge profile of the Li│in situ PTMC electrolyte│LFP cell at 0.1 C.


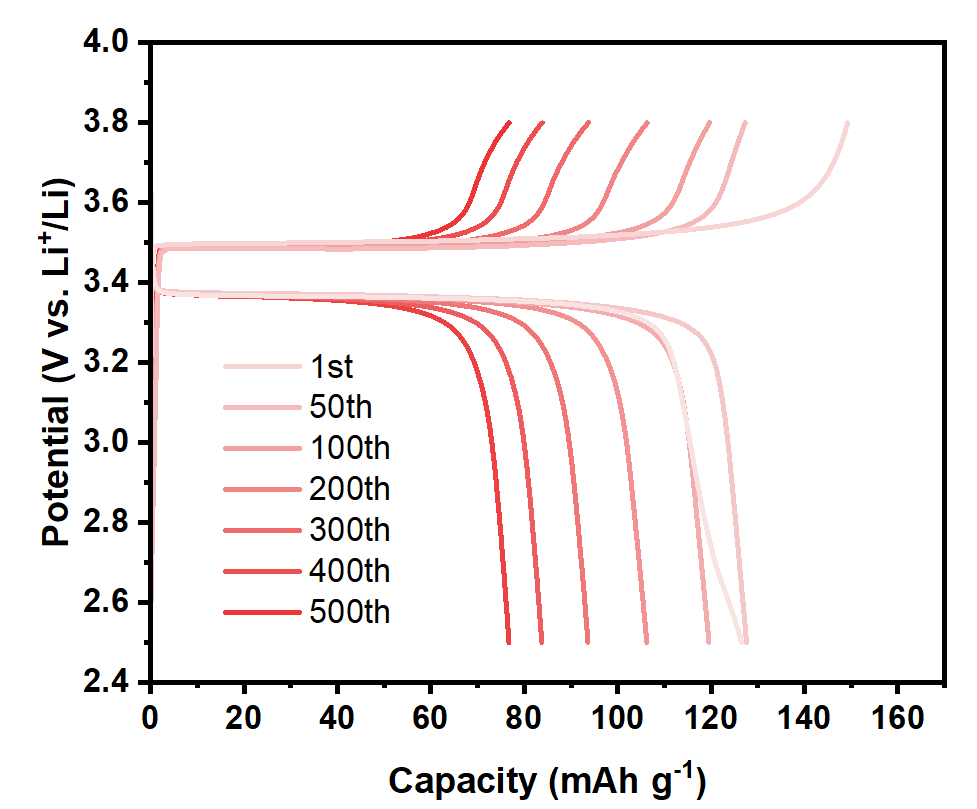


**Fig. S14**. Charge/discharge profile of the Li│TMC-LiDFOB│LFP cell at 1 C.


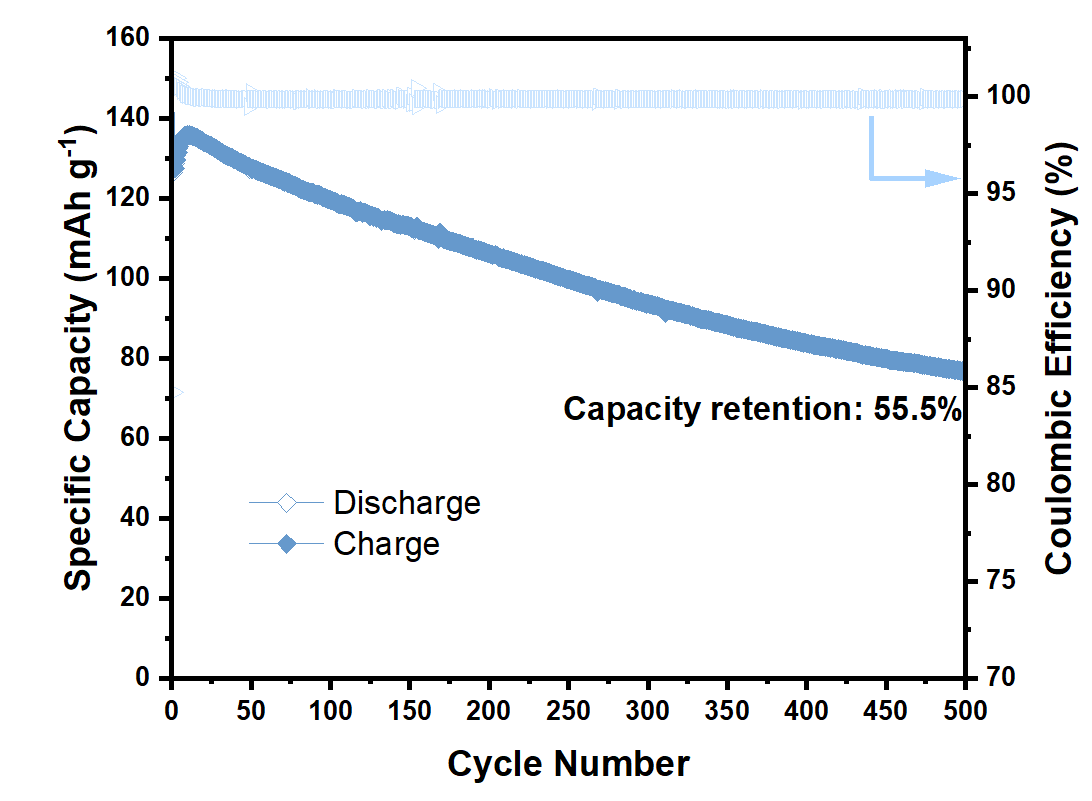


**Fig. S15**. Galvanostatic cycling performances of the unpolymerized TMC and LiDFOB liquid precursor at 1 C.


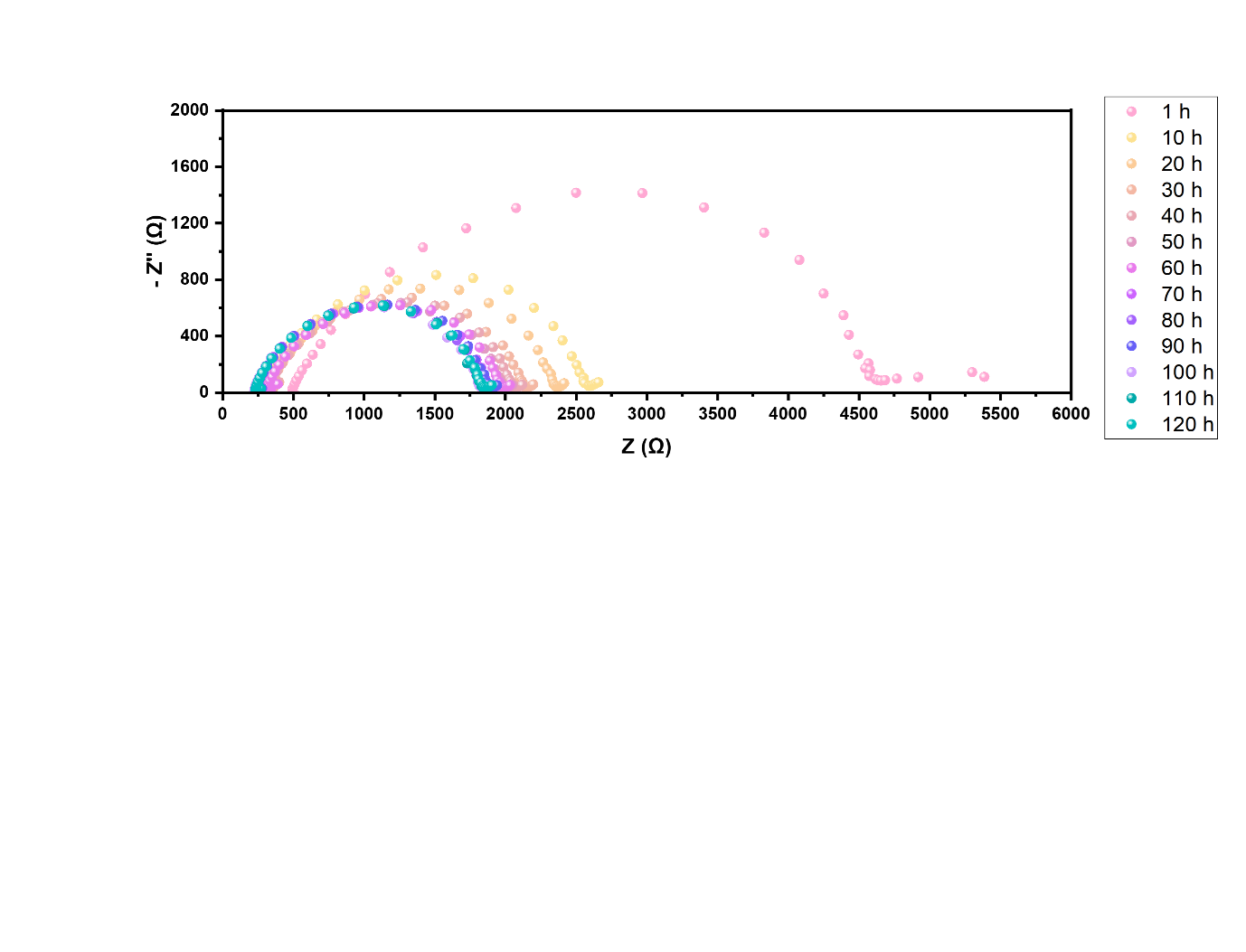


**Fig. S16.** Electrochemical impedance spectra of symmetric cell (Li│in situ PTMC electrolyte│Li) as a function of time.

# 6. Surface characterisation of cycled lithium anode

To further rationalise the observed cycling performance, the surface of a cycled lithium metal anode was characterised using FIB-SEM, EDX and XPS. The lithium metal displayed almost the same colour and a flat morphology after 50 cycles at 1C, and the presence of a relatively thick surface layer (1.5 µm). EDS mapping confirms that the layer contains phosphorus, fluorine, and boron species (Figure S16). N 1s and P 2p XPS spectra of the surface electrolyte layer and catalyst are identical (same binding energies and peak widths, Figures S18 and S19), confirming that nitrogen and phosphorus species retain the same oxidation state and tentatively suggesting that the phosphorus species detected by EDS are residual P2-tBu catalyst. Furthermore, common decomposition products such as LiF and Li_2_CO_3_, as well as boron-containing species (Li_x_BO_y_F_z_) were observed.


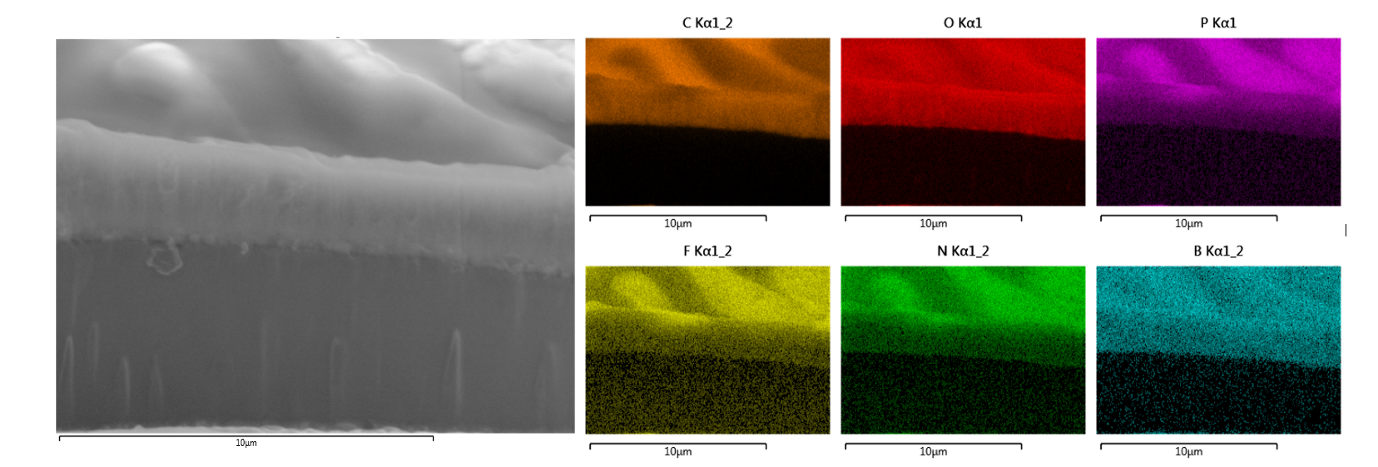


**Fig. S17.** FIB-SEM images of surface layer formed on Li cycled in in situ PTMC electrolytes.


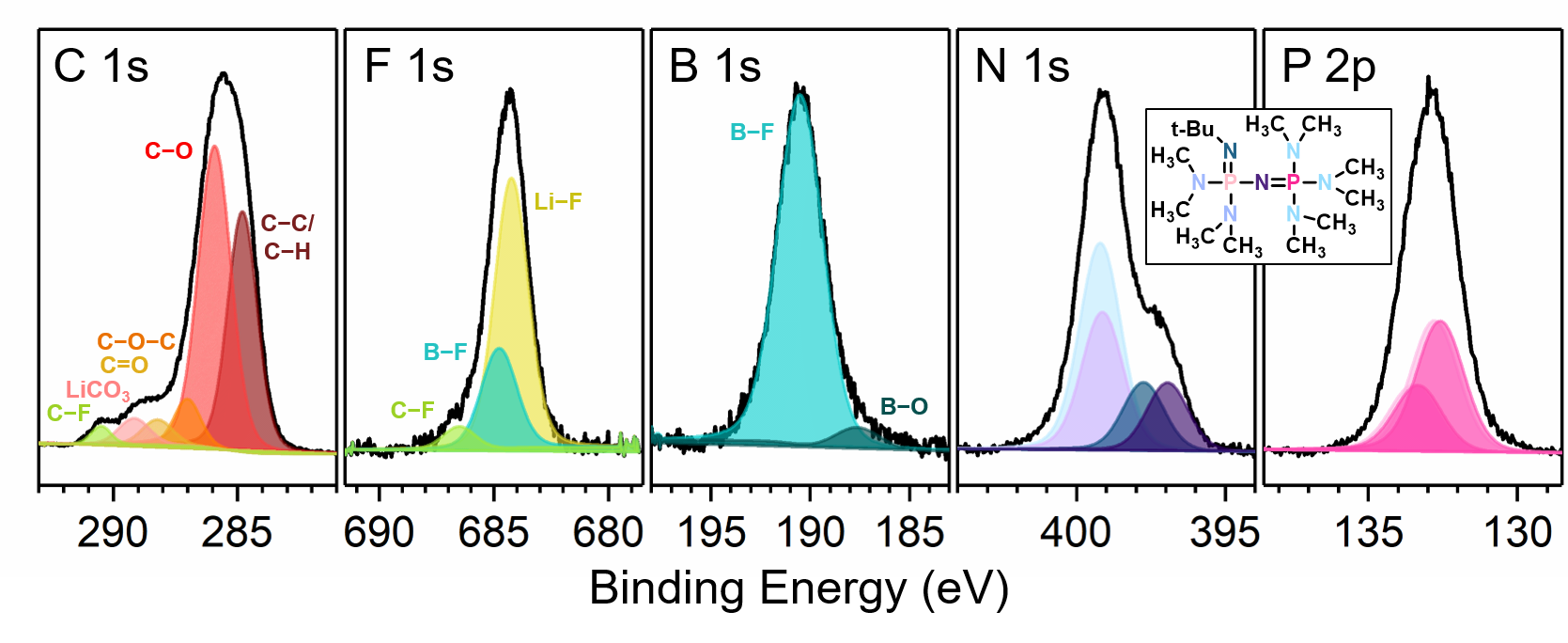


**Fig. S18**. XPS data of the surface layer onto Li metal after cycling in contact with the in situ PTMC electrolyte. Inset: structure of the P_2_-*t*Bu catalyst.


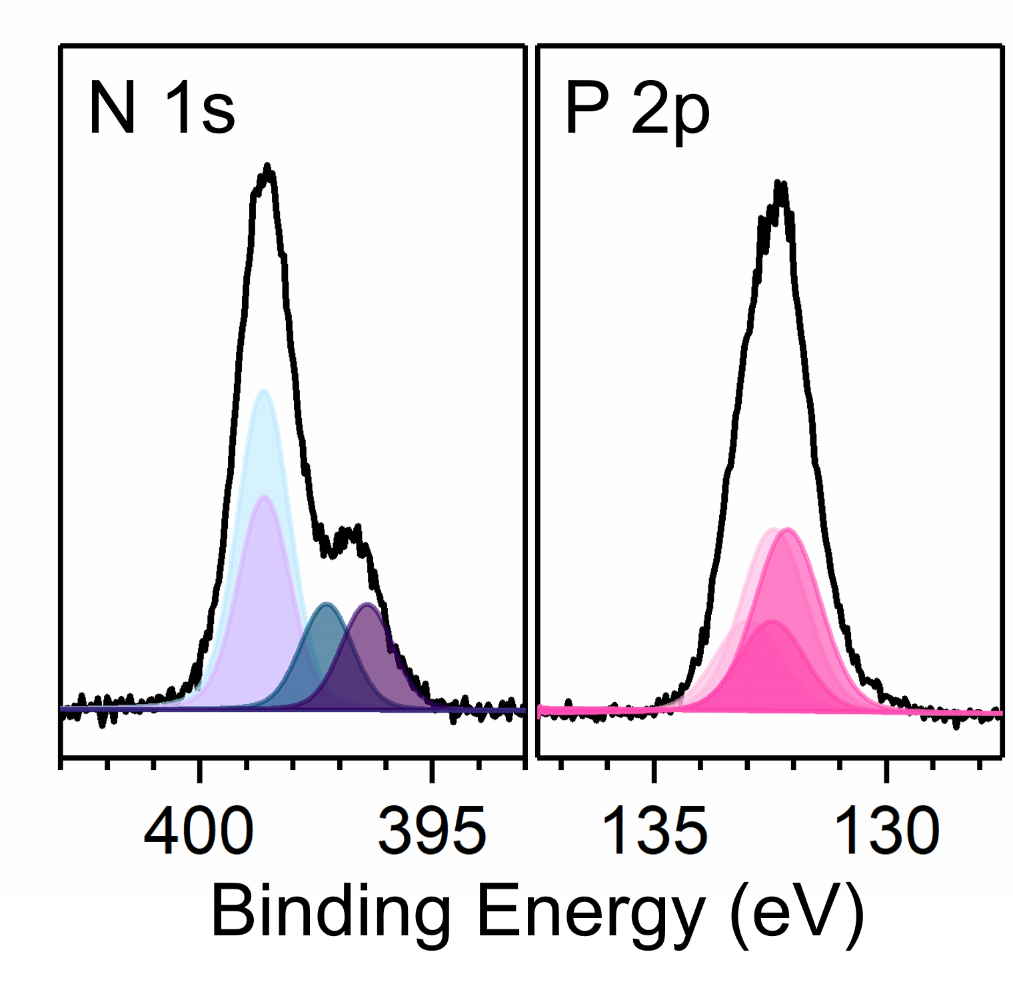


**Fig. S19.** XPS data of precipitated P_2_-*t*Bu catalyst.

# 7. Solid-state Recycling

**Scheme S2**. Proposed mechanism for the depolymerisation of TMC by ZnCl_2_/GEO.


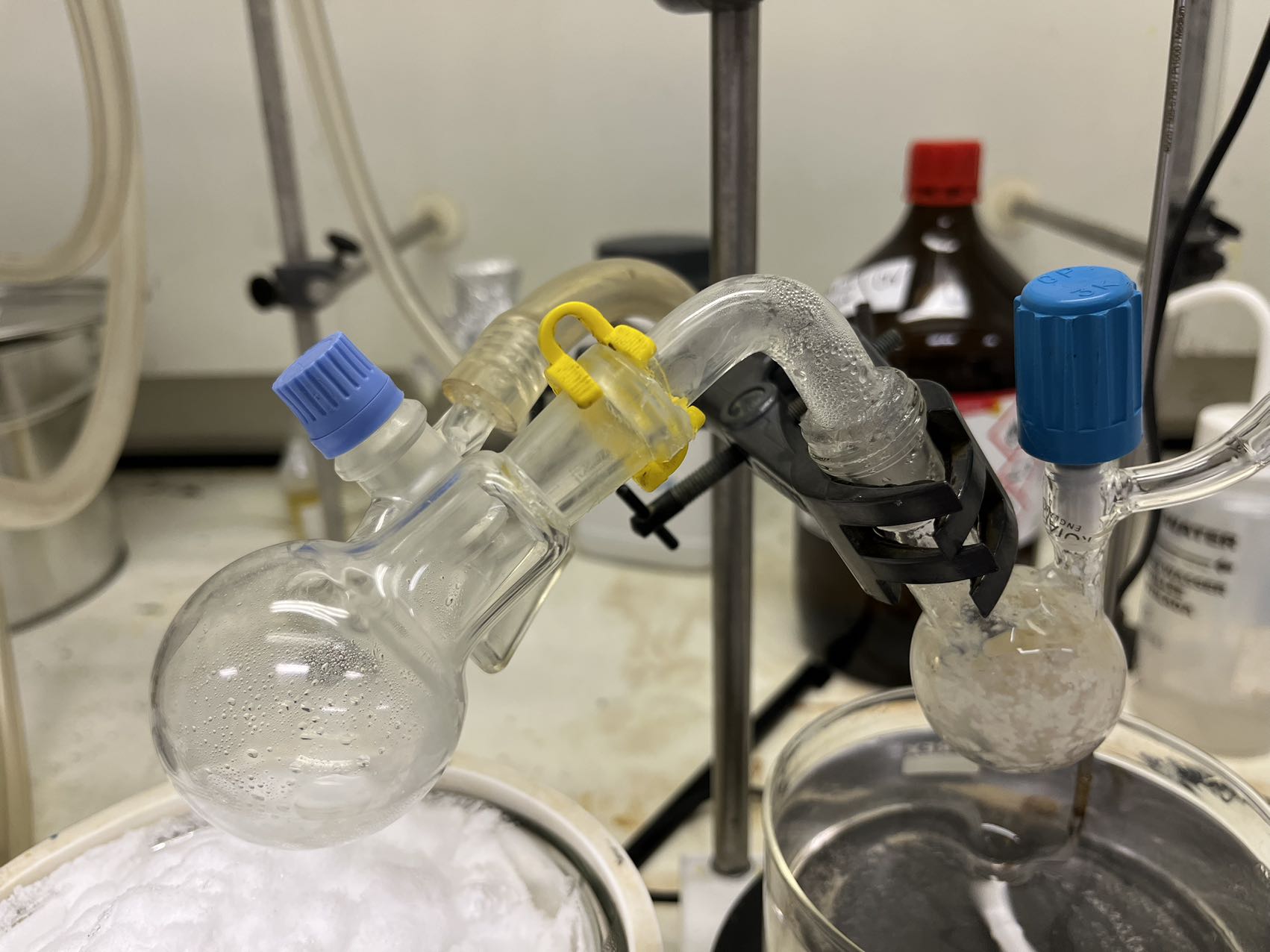


**Fig. S20.** The set-up of the solid-state depolymerization reaction.


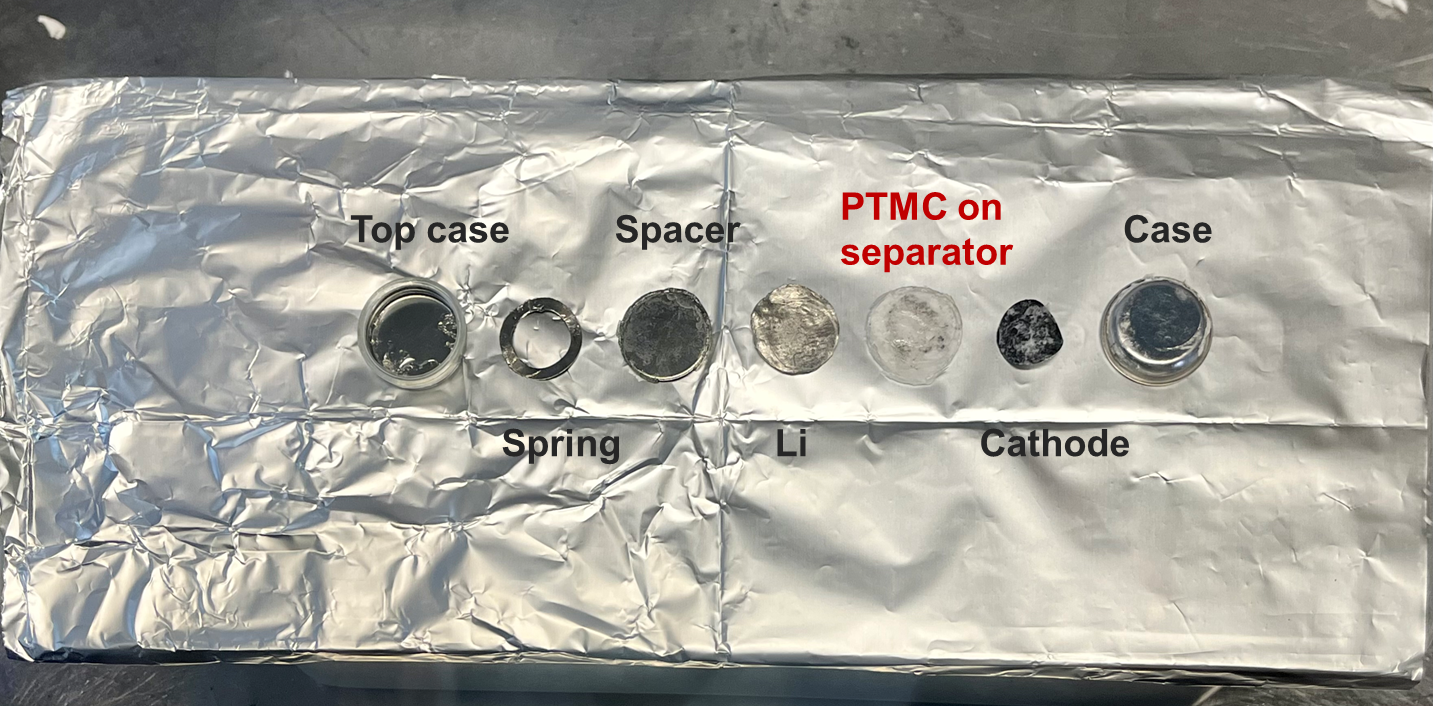


**Fig. S21.** Disassembled coin-cell after long-term galvanostatic cycling. PTMC electrolyte contained in glass fiber is extracted and used in solid-state recycling.

**Fig. S22**. ^1^H NMR spectrum (400 MHz, CDCl3, 295K) of recycled TMC. The recycling experiment results in minor impurity signals tentatively assigned to oligocarbonates. The TMC was used to make a recycled electrolyte without further purification and there was no compromise (from the baseline signals) to the polymerization or polymer electrolyte properties in the cell.

# 8. Comparison to Literature


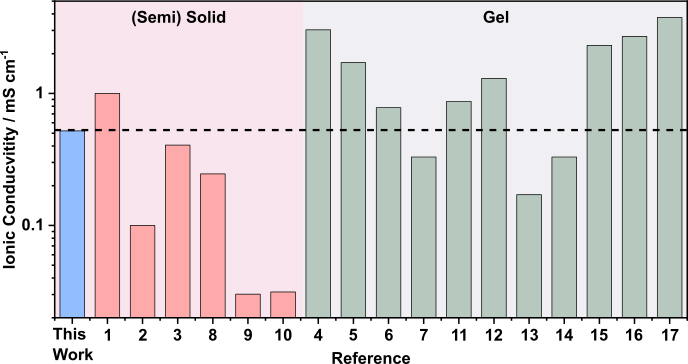


**Fig. S23.** Ionic conductivities of in situ polymer electrolytes reported in literature divided by gel (liquid content > 50%) and semi-solid and compared to this work. All have been measured at 23 – 30 °C , see Table S2 for values and polymer chemistries. The reference numbers correspond to the first column in Tables S2 / S3.


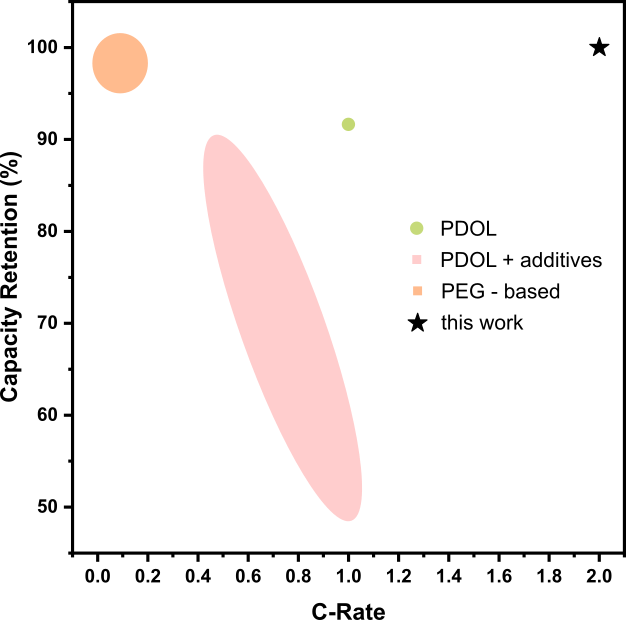


**Fig. S24**. Ashby plot of capacity retention performance vs. C-rate after 100 cycles (semi-solid electrolytes only). All entries are for LiFePO_4_ | polymer electrolyte | Li cells measured at 25 – 30 °C, see Table S3 for values and polymer chemistries. PDOL values are taken from ref. 1 in Table S3, PDOL + additives correspond to ref. 2 and 3 and PEG-based are ref. 9 and 10. The reference numbers correspond to the first column in Tables S2 / S3.





**Figure S25.** Scatter plot comparing capacity retention vs. C-rate after 350 cycles for semi-solid polymer electrolytes cycled on LiFePO_4_ | polymer electrolyte | Li cells and measured at 25 – 30 °C, see Table S3 for values and polymer chemistries (entries 1 and 3, Table S3). The reference numbers correspond to the first column in Tables S2 / S3.


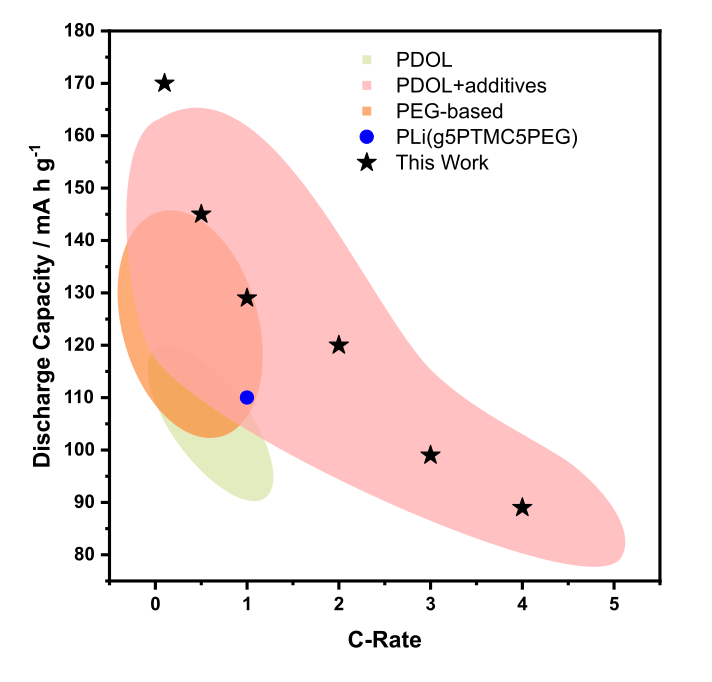


**Fig. S26**. Ashby plot of discharge capacity vs. C-rate. Semi-solid polymer electrolytes are compared. All entries are for LiFePO_4_ | polymer electrolyte | Li cells measured at 25 – 30 °C, see Table S3 for values and polymer chemistries. PDOL values are taken from ref. 1 in Table S3, PDOL + additives correspond to ref. 2 and 3 and PEG-based are ref. 9 and 10. The reference numbers correspond to the first column in Tables S2 / S3.

**Table S2**. Comparison of electrolyte properties for the in situ PTMC electrolyte and reported polymer electrolytes. (*) Ionic conductivities measured at 25 – 30 °C. (**) Oxidative stability measured in Li metal – Stainless Steel cells unless otherwise stated. Semisolid and solid electrolytes are marked in blue.

| **Reference** | **Polymer** | **Amount of solvent** | **Lithium salt** | **Ionic conductivity / mS cm^-1^ *** | **Oxidative stability / V **** | ***T*_d_ / °C** | **Ref.** |
| --- | --- | --- | --- | --- | --- | --- | --- |
| 1 | **Poly(DOL)** | Trace DOL | LiTFSI | 1.00 | 4.7 (Li \| PE \| NMC622) | 100 – 70 | ^[2]^ |
| 2 | **PDOL + succinonitrile** | Up to 30 % wt. SN | LiDFOB + LiTFSI | 0.1 | 5.1 | 104 | ^[3]^ |
| 3 | **Poly(DOL-TXE) TXE=1,3,5-trioxolane + succinonitrile** | DOL:TXE:SN 4:1:1 wt ratio conversion unknown | 1 : 1 LiTFSI : LiDFOB | 0.406 | 5.1 | 120 | ^[4]^ |
| 4 | **Poly(DOL) in EC / DEC / DMC** | 50 % DOL 50 % carbonate v/v | LiPF_6_ | 3.03 | 4.6 | - | ^[5]^ |
| 5 | **P(DOL) + PEG in DME** | 1 : 1 DOL : DME v/v | LiPF_6_ + LiTFSI | 1.71 | 4.4 | - | ^[6]^ |
| 6 | **P(DOL) in DME/DOL + LiNO3/FEC** | 10 : 85 : 85 FEC : DOL : DME v/v/v | LiPF_6_ + LiTFSI | 0.78 | 5 | - | ^[7]^ |
| 7 | **Crosslinked PDOL - glycerol triglycidyl ether (GTE) network plasticised with FEC, EMC and HFE** | 4 : 1 : 2 : 2 DOL : GTE : HFE : FEC : EMC v/v | LiTFSI + LiDFOB | 0.33 | 4.6 | 118 | ^[8]^ |
| 8 | **Poly(Pli)-g-5 PTMC/ 5 PEG / 0.5 TFE** PTMC = Polytrimethylenecarbonate and PEG = Polyethyleneglycol TFE = 2,2,2-trifluoroethoxy and PLi = lithium (2-hydroxymethyl) acrylate - co - lithium 2((2-bromoisobutyryloxy)methyl) acrylate) | Dry | LiTFSI | 0.246 | 5.3 | 240 | ^[9]^ |
| 9 | **Poly(ethylene glycol dimethacrylate-1,2-ethanedithiol)** | Dry | LiTFSI | 0.0302 | 4.5 | 320 | ^[10]^ |
| 10 | **Poly(ethylene glycol) methyl ether acrylate - *b* -caprolactone) “P(EGA-b-CL)” (30 wt. % precursor solution)** | Dry | LiTFSI | 0.0314 | 5.3 | 253 - 263 | ^[11]^ |
| 11 | **poly(polyethyleneglycol diacrylate-co-pentaerythritol triacrylate-co-polypropilene glycol) in EMC/FEC** | Gel unkown concentration | LiPF_6_ | 0.87 | 4.5 | 100 | ^[12]^ |
| 12 | **poly(ethyleneglycol methacrylate) poly(hexamethyene diisocyanate) vinylidene carbonate crosslinked network in EC / DEC + LiNO3** | 80 % wt. liquid electrolyte | LiPF_6_ | 1.3 | 4.8 | 111 | ^[13]^ |
| 13 | **vinyl ethylene carbonate - 2,2,2-trifluoroethyl methacrylate - polyethylene glycol dimethacrylate crosslinked network** | 10 : 5 VEC:TFEMA wt ratio (maximum concentration polymer) | LiTFSI | 0.171 | 5 | 130 | ^[14]^ |
| 14 | **poly(ethylene glycol) diacrylate / pentaerythritol tetra (3-mercaptopropionate) crosslinked network in deep eutectic solution of 1,3-diamino-2-propanol and sulfolane** | 60 - 80 wt % liquid | LiTFSI + LiDFOB | 0.33 | 4.8 | 150 | ^[15]^ |
| 15 | **Poly(hexamethylene diisocyanate) + FEC additive in EC / DEC** | Gel unkown concentration | LiPF_6_ | 2.31 | - | 81 | ^[16]^ |
| 16 | **Poly(ethyl cyanoacrylate) in EC / DMC** | 1 : 3 v/v monomer to liquid | LiClO_4_ | 2.7 | 4.8 | - | ^[17]^ |
| 17 | **Poly(1,3,5-trioxolane) in FEC/FEMC** | ca. 1g TXE in 2 mL of FEC / FEMC | LiFSI+LiDFOB+LiPF_6_ | 3.76 | 6.1 | - | ^[18]^ |
| 18 | **Poly(trimethylene carbonate) in TMC** | 93 mol % PTMC | LiTFSI (10 mol%) | 1.62 | 4.7 | 245 | ^[19]^ |
| 19 | **Poly(trimethylene carbonate) in TMC** | 63 mol % PTMC | LiDFOB (10 mol%) | 0.52 | 4.7 | 160 | **This work** |

**Table S3**. Comparison of battery cycling performance for the in situ PTMC electrolyte and reported polymer electrolytes. All entries in this table are for LiFePO_4_ | polymer electrolyte | Li cells.

| **Reference** | **Polymer** | **Mass loading / mg cm^-2^** | **Cycling rate (C)** | **Initial Discharge Capacity / mA h g^-1^** | **Number of Cycles** | **Capacity Retention (%)** | **Temperature / °C** | **Potential Window / V** | **Ref.** |
| --- | --- | --- | --- | --- | --- | --- | --- | --- | --- |
| 1 | **Poly(DOL)** | 5 | 1 | 95 | 700 | 80 | 25 | 2.5 - 4 | ^[2]^ |
|  |  | 5 | 0.2 | 115 | 200 | 83 | 25 | 2.5 - 4 |  |
| 2 | **PDOL + succinonitrile** | 2 | 1 | 107 | 100 | 50 | 25 | 2.4-4.2 | ^[3]^ |
|  |  |  | 0.1 | 134 |  |  |  |  |  |
|  |  |  | 0.2 | 141 |  |  |  |  |  |
|  |  |  | 0.5 | 132 |  |  |  |  |  |
|  |  |  | 2 | 109 |  |  |  |  |  |
|  |  |  | 3 | 96 |  |  |  |  |  |
|  |  |  | 5 | 82 |  |  |  |  |  |
| 3 | **Poly(DOL-TXE) TXE=1,3,5-trioxolane + succinonitrile** | 1.5-1.8 | 0.5 | 153 | 900 | 84 | 25 | 2.7 – 3.9 | ^[4]^ |
|  |  |  | 0.1 | 159 |  |  |  |  |  |
|  |  |  | 0.2 | 155 |  |  |  |  |  |
|  |  |  | 1 | 148 |  |  |  |  |  |
|  |  |  | 2 | 133 |  |  |  |  |  |
| 4 | **Poly(DOL) in EC / DEC / DMC** | 4.5 | 0.5 | 150 | 350 | 83 | 25 | 2.5 - 4 | ^[5]^ |
| 5 | **P(DOL) + PEG in DME** | 3 | 0.5 | 163 | 1000 | 77 | 25 | 4.2-2.7 | ^[6]^ |
|  |  |  | 0.2 | 161 | - |  |  |  |  |
|  |  |  | 1 | 150 | - |  |  |  |  |
|  |  |  | 2 | 139 | - |  |  |  |  |
|  |  |  | 5 | 118 | - |  |  |  |  |
| 6 | **P(DOL) in DME/DOL + LiNO3/FEC** | 4 | 0.1 | 168 | - | - | 25 | 2.0-4.0 | ^[7]^ |
|  |  |  | 0.2 | 158 | - | - |  |  |  |
|  |  |  | 0.5 | 159 | 200 | 90 |  |  |  |
|  |  |  | 1 | 130 | - | - |  |  |  |
|  |  |  | 2 | 133 | 1000 | 76 |  |  |  |
|  |  |  | 5 | 67 | - | - |  |  |  |
| 7 | **Crosslinked PDOL - glycerol triglycidyl ether (GTE) network plasticised with FEC, EMC and HFE** | 2 | 1 | 132 | 1000 | 88 | 25 | 4-2.5 | ^[8]^ |
|  |  | - | 2 | 120 | 500 | 90 |  |  |  |
| 8 | **Poly(Pli)-g-5 PTMC/ 5 PEG / 0.5 TFE** PTMC = Polytrimethylenecarbonate and PEG = Polyethyleneglycol TFE = 2,2,2-trifluoroethoxy and PLi = lithium (2-hydroxymethyl) acrylate - co - lithium 2((2-bromoisobutyryloxy)methyl) acrylate) | - | 2 | 130 | 1000 | 76 | 60 | 2.5-4.2 | ^[9]^ |
| 9 | **Poly(ethylene glycol dimethacrylate-1,2-ethanedithiol)** | 1.8 | 0.05 | 140 | 100 | 96 | 25 | 2.5 – 4 | ^[10]^ |
|  |  |  | 0.1 | 125 |  |  |  |  |  |
|  |  |  | 0.2 | 111 |  |  |  |  |  |
| 10 | **Poly(ethylene glycol) methyl ether acrylate - *b* -caprolactone) “P(EGA-b-CL)” (30 wt. % precursor solution)** | 1.5 - 2.0 | 0.1 | 137 | 180 | 96 | 30 | 2.5-4.2 | ^[11]^ |
|  |  | 1.5 - 2.0 | 0.2 | 136 | 5 | - | 30 | 2.5-4.2 |  |
|  |  | 1.5 - 2.0 | 0.5 | 125 | 5 | - | 30 | 2.5-4.2 |  |
|  |  | 1.5 - 2.0 | 1 | 109 | 5 | - | 30 | 2.5-4.2 |  |
| 11 | **poly(polyethyleneglycol diacrylate-co-pentaerythritol triacrylate-co-polypropilene glycol) in EMC/FEC** | 3.5 | 0.1 | 160 | - | - | 25 | 4-2.5 | ^[12]^ |
|  |  |  | 0.2 | 153 | - | - |  |  |  |
|  |  |  | 0.5 | 140 | - | - |  |  |  |
|  |  |  | 1 | 126 | 800 | 90 |  |  |  |
|  |  |  | 2 | 108 | - | - |  |  |  |
|  |  |  | 5 | 66 | - | - |  |  |  |
| 12 | **poly(ethyleneglycol methacrylate) poly(hexamethyene diisocyanate) vinylidene carbonate crosslinked network in EC / DEC + LiNiO3** | 3.5 | 1 | 147 | 850 | 96.5 | 30 | 2.5 - 4 | ^[13]^ |
|  |  |  | 0.1 | 167 |  |  |  |  |  |
|  |  |  | 0.2 | 166 |  |  |  |  |  |
|  |  |  | 0.5 | 160 |  |  |  |  |  |
|  |  |  | 2 | 120 |  |  |  |  |  |
| 13 | **vinyl ethylene carbonate - 2,2,2-trifluoroethyl methacrylate - polyethylene glycol dimethacrylate crosslinked network** | - | 0.1 | 158 | 200 | 81 | 25 | 4-2.5 | ^[14]^ |
| 14 | **poly(ethylene glycol) diacrylate / pentaerythritol tetra (3-mercaptopropionate) crosslinked network in deep eutectic solution of 1,3-diamino-2-propanol and sulfolane** | 2.0 - 3.0 | 0.5 | 120 | 28 |  | 25 | 2.5-4.2 | ^[15]^ |
|  |  |  | 1 | 96 |  |  |  |  |  |
|  |  |  | 2 | 74 |  |  |  |  |  |
|  |  |  | 5 | 48 |  |  |  |  |  |
|  |  |  | 10 | 27 |  |  |  |  |  |
| 15 | **Poly(hexamethylene diisocyanate) + FEC additive in EC / DEC** | 3 | 1 | 110 | 800 | 91 | 25 | ? | ^[16]^ |
|  |  |  | 0.2 | 131 | 5 | - | 25 | ? |  |
|  |  |  | 0.5 | 123 | 5 | - | 25 | ? |  |
|  |  |  | 2 | 88 | 5 | - | 25 |  |  |
| 16 | **Poly(ethyl cyanoacrylate) in EC / DMC** | - | 1 | 140 | 100 | 90 | 25 | 2.5-4 | ^[17]^ |
|  |  |  | 0.2 | 143 |  |  |  |  |  |
|  |  |  | 0.5 | 120 |  |  |  |  |  |
|  |  |  | 2 | 93 |  |  |  |  |  |
|  |  |  | 3 | 78 |  |  |  |  |  |
| 17 | **Poly(1,3,5-trioxolane) in FEC/FEMC** | - | 1 | 141 | 600 | 90 | 25 | 4-2.5 | ^[18]^ |
|  |  |  | 2 | 122 | 600 | 99 |  |  |  |
|  |  |  | 0.1 | 153 | - |  |  |  |  |
|  |  |  | 0.2 | 153 | - |  |  |  |  |
|  |  |  | 0.5 | 147 | - |  |  |  |  |
|  |  |  | 3 | 111 | - |  |  |  |  |
| 18 | **Poly(trimethylene carbonate) in TMC** | 10 | 0.2 | 143 | 85 | 92 | 40 | 3.5-2.5 | ^[19]^ |
| 19 | **Poly(trimethylene carbonate) in TMC** | 1 | 2 | 120 | 350 | 97 | 30 | 3.8-2.5 | **This work** |
|  |  |  | 0.1 | 170 | 35 | 95 |  |  |  |
|  |  |  | 0.5 | 145 |  |  |  |  |  |
|  |  |  | 1 | 129 |  |  |  |  |  |
|  |  |  | 3 | 99 |  |  |  |  |  |
|  |  |  | 4 | 89 |  |  |  |  |  |

# 9. References

[1] G. L. Gregory, G. S. Sulley, J. Kimpel, M. Łagodzińska, L. Häfele, L. P. Carrodeguas, C. K. Williams, *Angew. Chem. Int. Ed.* **2022**, *61*, e202210748.

[2] Q. Zhao, X. Liu, S. Stalin, K. Khan, L. A. Archer, *Nat. Energy* **2019**, *4*, 365-373.

[3] Q. Liu, B. Cai, S. Li, Q. Yu, F. Lv, F. Kang, Q. Wang, B. Li, *J. Mater. Chem. A* **2020**, *8*, 7197-7204.

[4] Z. Ren, J. Li, M. Cai, R. Yin, J. Liang, Q. Zhang, C. He, X. Jiang, X. Ren, *J. Mater. Chem. A* **2023**, *11*, 1966-1977.

[5] Q. Ma, J. Yue, M. Fan, S. J. Tan, J. Zhang, W. P. Wang, Y. Liu, Y. F. Tian, Q. Xu, Y. X. Yin, Y. You, A. Luo, S. Xin, X. W. Wu, Y. G. Guo, *Angew. Chem. Int. Ed.* **2021**, *60*, 16554-16560.

[6] Z. Wen, Z. Zhao, L. Li, Z. Sun, N. Chen, Y. Li, F. Wu, R. Chen, *Adv. Funct. Mater.* **2021**, *32*.

[7] X. Jiao, J. Wang, G. Gao, X. Zhang, C. Fu, L. Wang, Y. Wang, T. Liu, *ACS Appl. Mater. Interfaces* **2021**, *13*, 60054-60062.

[8] D. Yang, Y. Yang, Y. Cui, Y. Sun, T. Zhang, *J. Mater. Chem. A* **2024**, *12*, 27043-27052.

[9] K. Guo, S. Li, J. Wang, Z. Shi, Y. Wang, Z. Xue, *ACS Energy. Lett.* **2024**, *9*, 843-852.

[10] R. Xu, B. Xiao, C. Xuan, S. Gao, J. Chai, S. Liu, Y. Chen, Y. Zheng, X. Cheng, Q. Guo, Z. Liu, *ACS Appl. Mater. Interfaces* **2021**, *13*, 34274-34281.

[11] K. Guo, J. Wang, Z. Shi, Y. Wang, X. Xie, Z. Xue, *Angew. Chem. Int. Ed.* **2023**, *62*, e202213606.

[12] F. Wang, H. Liu, Y. Guo, Q. Han, P. Lou, L. Li, J. Jiang, S. Cheng, Y. Cao, *Energy Environ. Mater.* **2023**, *7*.

[13] J. Zheng, L. Duan, H. Ma, Q. An, Q. Liu, Y. Sun, G. Zhao, H. Tang, Y. Li, S. Wang, Q. Xu, L. Wang, H. Guo, *Energy Environ. Sci.* **2024**, *17*, 6739-6754.

[14] J. Xu, Y. Hu, M. Zhang, J. Cao, M. Wang, B. Hong, Y. Lai, *ACS Appl. Energy Mater.* **2024**, *7*, 10777-10783.

[15] W. Ye, J. Wang, C. Zhang, Z. Xue, *Energy Environ. Mater.* **2023**, *6*.

[16] Q. Hao, J. Yan, Y. Gao, F. Chen, X. Chen, Y. Qi, N. Li, *ACS Appl. Mater. Interfaces* **2024**, *16*, 44689-44696.

[17] Y. Cui, J. Chai, H. Du, Y. Duan, G. Xie, Z. Liu, G. Cui, *ACS Appl. Mater. Interfaces* **2017**, *9*, 8737-8741.

[18] R. He, K. Deng, T. Guan, F. Liang, X. Zheng, M. Li, D. Mo, K. Yang, H. Xie, *J. Colloid Interface Sci.* **2023**, *644*, 230-237.

[19] P. Chen, S. Liu, H. Zhou, S. Yan, D. Zhang, X. Pang, X. Chen, X. Wang, *J. Am. Chem. Soc.* **2025**, *147*, 7624-7633.
